# Occurrence, Fate, and Removal of Per- and Polyfluoroalkyl Substances (PFAS) in Small- and Large-Scale Municipal Wastewater Treatment Facilities in the United States

**DOI:** 10.1021/acsestwater.4c00541

**Published:** 2024-11-15

**Authors:** Juhee Kim, Xiaoyue Xin, Gary L. Hawkins, Qingguo Huang, Ching-Hua Huang

**Affiliations:** † School of Civil and Environmental Engineering, Georgia Institute of Technology, Atlanta, Georgia 30332, United States; ‡ Department of Crop and Soil Sciences, University of Georgia, Athens, Georgia 30223, United States; § Department of Crop and Soil Sciences, University of Georgia, Griffin, Georgia 30223, United States; ∥ Department of Civil, Environmental and Construction Engineering, University of Hawai′i at Ma̅noa, Honolulu, Hawaii 96822, United States

**Keywords:** PFAS, small- and large-scale wastewater treatment plants, municipal wastewater, perfluoroalkyl acids, precursors, TOP assay

## Abstract

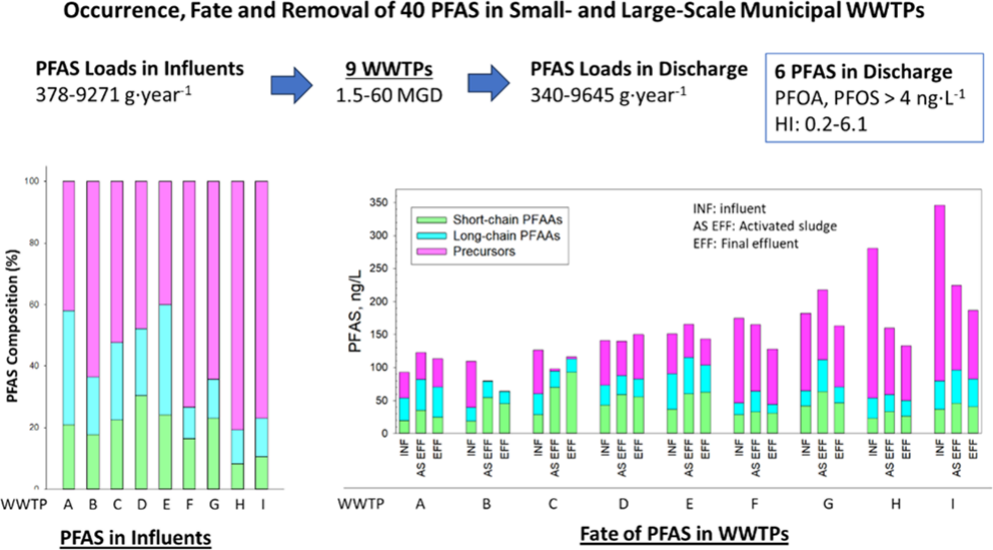

Wastewater treatment plants (WWTPs) could be conduits of polyfluoroalkyl substances (PFAS) contaminants in the environment. This study investigated the fate of 40 PFAS compounds across nine municipal WWTPs with varying treatment capacity and processes. High concentrations of perfluoroalkyl carboxylic acids (PFCAs) and perfluoroalkyl sulfonic acids (PFSAs) were detected in wastewater, with the ratio of their total concentrations (∑PFCAs/∑PFSAs) always greater than one. Transformation of precursors by activated sludge processes significantly increased the concentrations of short-chain PFCAs (e.g., perfluoropentanoic acid (PFPeA) and perfluorohexanoic acid (PFHxA)), while further advanced treatment processes offered minimal removal of perfluoroalkyl acids. Treatment capacity and PFAS removal efficiency showed no apparent correlation. The maximum possible PFAS loads discharged from WWTPs were 340–9645 g·year^–1^, similar to those entering the WWTPs. Among six regulated PFAS compounds, detection frequency was 100% for five (perfluorooctanoic acid (PFOA), perfluorooctanesulfonic acid (PFOS), perfluorononanoic acid (PFNA), perfluorobutanesulfonic acid (PFBS), and perfluorohexanesulfonic acid (PFHxS)) and 67% for hexafluoropropylene oxide dimer acid (HFPO–DA) (Gen-X). Concentrations of PFOA and PFOS in WWTP discharges consistently exceeded 4 ng·L^–1^. The hazard index (HI) for mixtures containing two or more of the four PFAS (PFNA, PFBS, PFHxS, and HFPO–DA) ranged from 0.2 to 6.1. These findings indicate that wastewater discharges may pose a risk, emphasizing the need for enhanced PFAS removal strategies in wastewater treatment processes.

## Abstract 2

Abundant short-chain PFCAs and PFOS are present in municipal wastewaters with limited removal at small- to large-scale treatment plants, while precursors in wastewater are transformed predominantly into short-chain PFCAs during activated sludge treatment.

## 1 Introduction

Per- and polyfluoroalkyl substances (PFAS) are classified as persistent organic pollutants known for their ability to accumulate in organisms and human bodies due to high biological resistance.^1−4^ PFAS are extensively used in many different consumer and industrial products. Scientific studies have demonstrated that exposure to certain PFAS is associated with adverse health effects including immune suppression, reduced fertility, and testicular and kidney cancer.^2,5−11,^ With increasing concerns over PFAS, the United Sates Environmental Protection Agency (U.S. EPA) recently finalized the National Primary Drinking Water Regulation (NPDWR) establishing legally enforceable levels (Maximum Contaminant Levels (MCLs)) for six PFAS compounds including perfluorooctanoic acid (PFOA), perfluorooctanesulfonic acid (PFOS), perfluorononanoic acid (PFNA), hexafluoropropylene oxide dimer acid (HFPO–DA, commonly known as GenX Chemical), and perfluorohexanesulfonic acid (PFHxS), and perfluorobutanesulfonic acid (PFBS).^12^

PFAS have been consistently detected in the aquatic environment, with typical concentrations of terminal perfluoroalkyl acids (PFAAs) ranging from pg·L^–1^ to ng·L^–1^.^13^ The point sources of PFAS include their manufacturing facilities and industrial sites where PFAS are used, such as airports, paper and pulp mills and textile mills.^14^ Municipal wastewater treatment plants (WWTPs) are also considered as point sources of PFAS to the aquatic environment due to inefficient removal of PFAS, despite not generating PFAS compounds.^15−22^ Perfluoroalkyl acid (PFAA) concentrations in wastewater effluents have been reported to be high up to several hundred ng·L^–1^.^23−27^ The high PFAA concentrations found in wastewater effluents are likely linked to the presence of precursor PFAS compounds in wastewater.^19^ Most of the precursor PFAS compounds are not included in the standard analytical methods utilized for regulatory purposes. These precursors have the potential to undergo transformation into terminal PFAAs in the environment and during (waste)water treatment processes.^19,28−32^ Not all precursors undergo the complete transformation to PFAAs, and PFAAs are not efficiently removed during treatment processes in WWTPs.

In the United States, approximately 15,000 municipal WWTPs are in operation, with 78% treating less than one million gallons per day (MGD).^33^ The majority of publicly owned WWTPs in each State, with a few exceptions, serve small communities, accounting for 80–95% of WWTPs.^34^ Moreover, in the past decade, 95% of nonmetropolitan counties in the U.S. experienced a growth rate of less than 10%, highlighting the trend of many of these small communities either slowly growing or declining in population.^35^ Rural and small-scale WWTPs are facing challenges with aging infrastructure.^36^ Typical full-scale wastewater treatment trains consist of the sequence of preliminary (e.g., screen) treatment, primary clarification, activated sludge, secondary clarification, and disinfection by chlorine, ultraviolet (UV) light, or ozone (O_3_). Large-scale WWTPs may operate further advanced treatment processes, including filtration, activated carbon, and membrane bioreactor. A full understanding of the fate of PFAS within the treatment processes and the potential transformation of precursors into more persistent PFAS remains limited, especially when considering the distinctions between small- and large-scale facilities and the various treatment processes employed.

To address this knowledge gap, this research aimed to achieve the following specific objectives to (i) investigate the occurrence of a broad range of PFAS (40 in total) and unknown precursors in wastewaters entering nine different small- to large-scale WWTPs in the U.S., (ii) assess the effectiveness of WWTPs equipped with different treatment processes, including conventional and various advanced treatment, in removing PFAS, and (iii) evaluate the fate of PFAS and precursors throughout the treatment processes in WWTPs. Grab or composite wastewater samples were collected from different stages within the treatment processes to analyze the trends in the fate of PFAS. Wastewater samples were analyzed by comprehensive methods, including both targeted analysis using authentic standards and the total oxidizable precursor (TOP) assay, to characterize the fate of 40 PFAS compounds and unknown PFAA precursors in the WWTPs.

## 2 Materials and Methods

### 2.1 Chemicals

This research investigated 40 PFAS (Supporting Information (SI) Table S1) including: 11 perfluorocarboxylic acids (**PFCAs**: PFBA, PFPeA, PFHxA, PFHpA, PFOA, PFNA, PFDA, PFUdA, PFDoA, PFTrDA, PFTeDA); 8 perfluoroalkyl sulfonic acids (**PFSAs**: PFPrS, PFBS, PFPeS, PFHxS, PFHpS, PFOS, PFNS, PFDS); 3 fluorotelomer sulfonic acids (**FTSs**: 4:2 FTS, 6:2 FTS, 8:2 FTS); 3 fluorotelomer (unsaturated) carboxylic acids (**FT(U)CAs**: 6:2 FTCA, 5:3 FTCA, 6:2 FTUCA); 3 perfluorosulfonamides (**FASAs**: FBSA, FHxSA, FOSA); 2 perfluorosulfonamidoacetic acids (**FASAAs**: *N*-MeFOSAA, *N*-EtFOSAA); 8 per- and polyfluoroethers (**PFEAs**: HFPO–DA, ADONA, 9Cl-PF3ONS, 11Cl-PF3OUdS, PFEESA, PF4OPeA, PF5OHxA, 3,6-OPFHpA); and 2 fluorotelomer phosphate diesters (**diPAPs**: 6:2 diPAP, 6:2/8:2 diPAP). Information on sources of PFAS chemicals, analytical standards, isotope-labeled surrogates, and other chemicals used in this study is provided in SI Text S1.

### 2.2 Wastewater Sample Collection

Wastewater samples were collected between September 2021 and August 2023 from nine municipal WWTPs located across the U.S. The 24-h composite samples were collected from seven WWTPs, while grabs samples were taken from the other two WWTPs (WWTP B and C). Composite samples provide an average PFAS concentration over 24 h, reflecting typical plant conditions, while grab samples capture point-in-time levels based on plant dynamics. These differing methods may influence PFAS concentration and composition.

Samples were collected from three stages of treatment processes, specifically from primary clarifier (INF), activated sludge process (AS), and final effluent (EFF). The permitted treatment capacity of the WWTPs ranged from 1.5 to 60 MGD. The treatment processes include: preliminary (screen, grit removal), primary clarifier, activated sludge biological treatment, secondary clarifier, ultrafiltration, granular media filter, membrane bioreactor, activated carbon filter, ozonation, chlorination, and UV disinfection. Detailed information on the treatment processes in each WWTP is provided in Table 1. Note that WWTPs are listed from A to I based on the order of PFAS concentrations in INF, from the lowest to highest.

**Table 1:** Wastewater Treatment Plants (WWTPs) Specifics

WWTPs	MGD^a^	treatment description	sampling date
A	60	screen; activated sludge reactor; chlorination	August 2023
B	22	screen; activated sludge reactor; membrane bioreactor; UV	September 2021
C	60	screen; activated sludge reactor; granular media filter; activated carbon filter; ozonation	September 2021
D	24	screen; activated sludge reactor; UV	May 2022
E	40	screen; activated sludge reactor; granular media filter; chlorination	April 2022
F	8	screen; activated sludge reactor; UV	April 2023
G	1.5	screen; activated sludge reactor; UV	March 2023
H	11.7	screen; activated sludge reactor; UV	November 2022
I	27	screen; activated sludge reactor; UV	February 2023

a Permitted MGD capacity.

Wastewater samples were carefully collected to minimize the risk of cross-contamination and false positives, based on the standard operating procedure for sampling.^37^ Briefly, PFAS-containing products or materials were consciously avoided. New nitrile gloves were utilized for each sampling event. Samples were collected in new 2 L high-density polyethylene (HDPE) bottles that were rinsed with methanol and high-purity deionized water (DI water, >18 mΩ·cm) in the laboratory, and were tightly sealed with HDPE screw caps. A field reagent blank (FRB) was included at each sample site to monitor potential contamination. All sampling materials were used only once to prevent cross-contamination. During transportation or shipping, samples were chilled with ice. Upon arrival at the laboratory, samples underwent centrifugation and vacuum-filtration through glass fiber filters (∼0.7 μm pore-size). Subsequently, these samples were stored at 4 °C for no more than 14 days before extraction.

### 2.3 Sample Pretreatment for Unknown Precursors

To estimate the levels of potential precursors in wastewater samples, the total oxidizable precursor (TOP) assay was employed for sample pretreatment.^38−40^ The TOP assay pretreatment oxidizes unknown precursors to PFAAs by hydroxyl and sulfate radicals, hence enabling the measurement of the concentration of unknown oxidizable PFAS that are not directly measurable. Briefly, sample aliquots (250.0 mL), 250.0 mL of 120.0 mM potassium persulfate, and 7.2 mL of 10 M NaOH were transferred to prerinsed 500 mL HDPE bottles (with minimum headspace). The mixtures were thoroughly agitated in the shaker and sonicated for 5 min. Subsequently, the bottles were placed in a temperature-controlled water bath at 80–85 °C for at least 12 h. Following cooling, mixtures were neutralized to achieve a pH range between 6.0 and 8.0 using 1.0 M HCl. Samples were then stored at 4 °C for a maximum of 2 days before extraction.

### 2.4 Solid-Phase Extraction

Wastewater samples and TOP samples underwent solid-phase extraction (SPE) using Phenomenex SPE cartridge (Cat. No. 8B–S038–HCH), based on the slightly modified EPA methods (EPA 533, 537.1, and 1633).^41−43^ The 40 PFAS were quantified by comparing the relative response of analytes to their respective isotope-labeled surrogates (SI Table S2). Duplicate aliquots of each sample (500.0 mL each) were spiked with isotope-labeled surrogate mixtures before the extraction process, with a spiking concentration set at 12.0 ng·L^–1^ (48.0 ng·L^–1^ for FTSs). SPE cartridges were preconditioned on an SPE manifold with methanol, phosphate buffer and DI water. Following SPE cartridge conditioning, samples and rinsates from sample bottles were loaded to SPE cartridges through polyethylene tubing. Dried cartridges were flushed with 10.0 mL of solution containing 2.0% ammonium hydroxide (v/v) in methanol, into 15 mL polypropylene (PP) tubes. Extracts were evaporated until nearly dry by using a vacuum concentrator, then reconstituted in 500.0 μL of 80/20 (v/v) methanol/H_2_O, vortexed, sonicated, sealed, and transferred to PP HPLC vials for storage at 4 °C until analysis by liquid chromatography time-of-flight mass spectrometry (LC-TOFMS).

### 2.5 LC-TOFMS Analysis

Extracted wastewater and TOP samples were introduced into an Agilent 1260 Infinity HPLC with 6230 TOFMS operating under electrospray ionization in negative (ESI^–^) ion mode. Full scan mass spectra were acquired across the range of 50–1000 *m*/*z* with a mass accuracy of within 10 ppm. The drying gas flow rate, gas temperature, nebulizer pressure, capillary voltage, and fragmentation voltage were maintained at 9 L·min^–1^, 200 °C, 40 psi, 4000 V, and 140–250 V, respectively. Mass accuracy was continuously adjusted using reference standards with reference masses of 119.036320 (purine) and 980.016375 (hexakis(3,3,2,2-tetrafluoropropoxy)cyclotriphosphazene, acetate adduct). Samples of 10.0 μL were injected and separated using a Poroshell 120 EC–C18 column (2.1 × 150 mm, 2.7 μm) within the chromatographic method featuring a multistep gradient spanning 33 min at a constant flow rate of 0.3 mL·min^–1^. The eluent comprised 5.0 mM ammonium acetate and 80:20 (v/v) methanol/acetonitrile. Following a 2 min hold at 100% of 5.0 mM ammonium acetate, the organic phase was increased to 60% over 3 min, to 98% over 10 min, followed by a 7 min hold at 98%. Subsequently, the gradient was ramped to 100% of 5.0 mM ammonium acetate, and a 10 min postrun equilibration period ensued. All peaks were well resolved. For PFHxS, PFOS, *N*-MeFOSAA, and *N*-EtFOSAA, both linear and branched isomers were quantified by summing all isomer peaks. Detailed information is provided in SI Table S2.

### 2.6 Analytical Quality Control (QC)

Detailed information on calibration curve, continuing calibration check, instrument blank, and QC samples is provided in SI Text S2 and Table S3.

## 3 Results and Discussion

### 3.1 Detected PFAS in Wastewater

The concentrations of PFAS in the influent wastewater (INF) entering nine WWTPs are outlined in SI Tables S4–S12. Out of the 40 PFAS analyzed, 28 were detected in the INF, above the method detection limit (MDLs), ranging from 14 to 21 detected per WWTP. The summed of detected PFAS concentrations in INF (∑PFAS_INF_) ranged from 93.2 ng·L^–1^ (WWTP A) to 346.2 ng·L^–1^ (WWTP I). The maximum possible PFAS loads entering WWTPs were estimated by multiplying ∑PFAS_INF_ by the permitted wastewater flow capacity (i.e., MGD). The PFAS loads ranged from 378 g·year^–1^ (WWTP G) to 9271 g·year^–1^ (WWTP C) (Table 2). Note that we categorized PFAS compounds into two groups: PFAAs (PFCAs and PFSAs) and known precursors (FTSs, FT(U)CAs, FASAs, FASAAs, diPAPs, and PFEAs).

**Table 2:** PFAS Loads (Inputs and Outputs; g·year^–1^)^a^ in WWTPs

	∑40 PFAS	PFOA	PFOS	PFNA	PFBS	PFHxS	HFPO–DA
Influent							
mean	5110.9	412.5	524.7	47.7	211.0	64.6	580.6
SD^b^	2989.6	418.7	774.8	51.0	194.7	60.2	774.8
median	4681.3	268.6	172.6	22.2	130.5	59.3	172.6
maximum	9271.2	1212.6	1443.7	133.6	601.8	184.8	1950.5
minimum	378.3	20.4	14.3	0.0	11.0	2.0	0.0
no. detection (*n*/9)		9/9	9/9	8/9	9/9	9/9	6/9
Effluent							
mean	4568.5	536.2	447.9	39.8	339.7	76.3	712.0
SD	3587.8	602.3	515.8	47.7	445.1	71.7	1004.9
median	3265.3	294.8	276.3	14.0	168.6	39.9	75.6
maximum	9644.7	1838.5	1643.4	122.8	1330.5	168.7	1832.7
minimum	339.5	22.5	13.8	0.9	30.4	3.2	0.0
no. detection (*n*/9)		9/9	9/9	9/9	9/9	9/9	6/9

a PFAS loads = [PFAS] × permitted MGD × 365.

b Standard deviation.

#### 3.1.1 PFAAs

**Figure 1:**
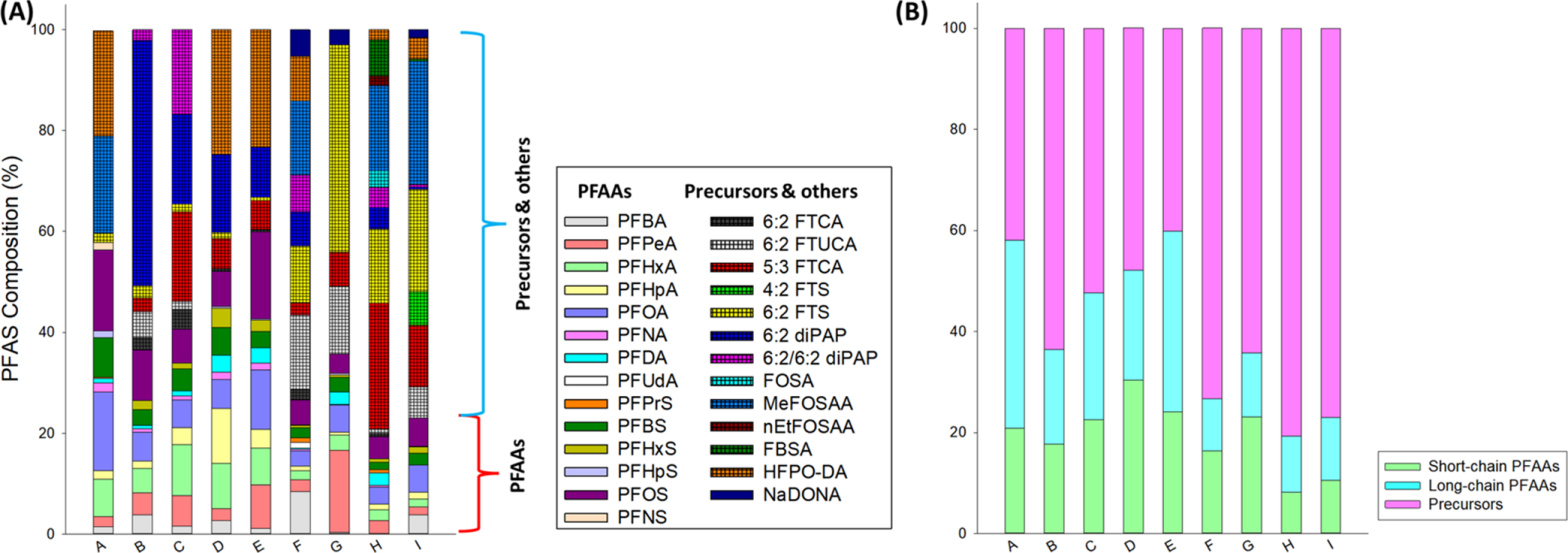
Percent composition of (A) individual PFAS (PFAAs, precursors and others) and (B) PFAS classes (short-chain PFAAs (PFCAs < C8, PFSAs < C6), long-chain PFAAs, precursors, and other PFAS) in nine WWTP influents (A–I).

Out of 19 PFAAs, 16 PFAAs were detected in INF, ranging from 9 to 14 detected per WWTP: PFBA, PFPeA, PFHxA, PFHpA, PFOA, PFNA, PFDA, PFUdA, PFDoA, PFPrS, PFBS, PFPeS, PFHxS, PFHpS, PFOS, and PFNS. The detection frequencies (in %) of individual PFAAs varied across the nine WWTPs, from 11% (PFUdA and PFPeS) to 100% (PFPeA, PFHxA, PFHpA, PFOA, PFBS, PFHxS, and PFOS). The detected PFAAs varied across the WWTPs and in concentrations. As shown in Figure 1A, PFOS was the most abundant in the INF of 5 WWTPs (A, B, E, H, and I). In WWTP C, PFHxA was the most prevalent compound, while PFHpA dominated in WWTP D, PFBA in WWTP F, and PFPeA in WWTP G. Concentrations of PFAAs in the INF across nine WWTPs followed this order (concentration values are indicated as average and median concentrations; Tables S4–S12): PFOS (13.1 and 11.0 ng·L^–1^) > PFOA (10.7 and 9.6 ng·L^–1^) > PFPeA (8.5 and 5.2 ng·L^–1^) ≈ PFHxA (7.4 and 6.0 ng·L^–1^) > PFBS (5.5 and 5.0 ng·L^–1^) > PFBA (4.6 and 1.8 ng·L^–1^) > PFHpA (4.2 and 3.1 ng·L^–1^) > PFDA (2.6 and 1.2 ng·L^–1^) ≈ PFHxS (2.4 and 1.8 ng·L^–1^) > PFNA (1.0 and 0.8 ng·L^–1^) > others.

The summed concentrations of PFAA (∑PFAA_INF_) in INF varied from 39.9 ng·L^–1^ (WWTP B) to 90.8 ng·L^–1^ (WWTP E). Concentrations of PFCAs (∑PFCA_INF_) ranged from 23.5 to 55.9 ng·L^–1^, while concentrations of PFSAs (∑PFSA_INF_) ranged from 13.6 to 34.8 ng·L^–1^. Short-chain (C4–C7 for PFCAs and C3–C5 for PFSA) and long-chain PFAAs (≥C8 for PFCAs and ≥C6 for PFSAs) accounted for 8.19–30.43 and 10.25–37.18% of the total PFAS in INF samples, respectively (Figure 1B). Average chain lengths of PFCA and PFSA were calculated using the following equation^44^
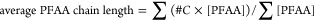
1where #*C* is the carbon number and [PFAA] is the concentration of the specific PFAA. For the nine WWTPs, the average PFCA chain length was found to be 6.57, and the average PFSA chain length was 6.61. Note that PFCAs with a carbon chain length of *C* ≥ 8 are considered long-chain, while for PFSAs, a chain length of *C* ≥ 6 is considered long-chain. This indicates that long-chain analogues were more dominant for PFSAs than for PFCAs in the municipal wastewater investigated in this study. This result is consistent with previous findings, indicating that even though PFOS has been phased out in the U.S. and Canada since 2000, PFOS is still frequently detected in municipal wastewaters.^44,45^ In addition, the ratio of ∑PFCA to ∑PFSA (∑PFCA/∑PFSA) was found to be greater than one (SI Figure S1), which is a typical trend observed in municipal wastewater.^46^

#### 3.1.2 Known Precursors

For known PFAS precursors (∑Precursor_INF_), which included FTSs, FT(U)CAs, FASAs, FASAAs, diPAPs, and PFEAs, their combined concentration ranged from 39.1 (WWTP A) to 266.5 ng·L^–1^ (WWTP I). Out of the 21 precursors, 13 precursors (6:2 FTCA, 6:2 FTUCA, 5:3 FTCA, 4:2 FTS, 6:2 FTS, 6:2 diPAP, 6:2/8:2 diPAP, FOSA, *N*-MeFOSAA, *N*-EtFOSAA, FBSA, HFPO–DA, and ADONA) were detected in INF, ranging from 3 to 11 detected per WWTP.

The most frequently detected known precursor was 6:2 FTS, which was detected in all nine WWTPs, followed by 5:3 FTCA (detected in 8 WWTPs) and 6:2 diPAP (detected in 7 WWTPs). 6:2 FTS and 6:2 diPAP are frequently detected in consumer products. For example, previous research reported that 6:2 FTS was found in all food packaging materials (*n* = 47) at concentrations ranging from 0.001 to 0.1 ng·g^–1^.^47^ Another research group found that 6:2 FTS and 6:2 diPAP were detected in cosmetics at detection rate of 56 and 84%, respectively, among the 45 cosmetic products tested.^48^ 6:2 diPAP can undergo microbial transformation to form 6:2 fluorotelomer alcohol (6:2 FTOH), which in turn can be further transformed to 5:3 FTCA.^49,50^ Meanwhile, 5:3 FTCA is also frequently detected in food packaging materials.^51−53^ Among 8 PFEAs, only HFPO–DA (detected in 5 WWTPs) and ADONA (detected in 3 WWTPs) were detected in INF. PFEAs are produced through fluorotelomerization and used as replacements for phased-out long-chain PFAAs. Several recent studies have reported an increased frequency and higher concentrations of HFPO–DA detected in municipal wastewater.^54,55^

#### 3.1.3 Impact of Industrial Point Sources

While the facilities sampled in this study were all municipal WWTPs, it is unclear whether significant point sources discharged PFAS into their municipal sewers. The presence of potential PFAS point sources near WWTPs has been investigated by other studies, including airports,^56^ fire training sites,^57,58^ and landfill sites.^59^ Potential point sources within the same county as the WWTPs in this study were: WWTP A (airport); WWTP B (closed landfill); WWTP C (active landfill, airport); WWTP D (active landfill, closed landfill); WWTP E (airport), and WWTP I (airport). There are at least >2000 active municipal landfills in the U.S.,^60^ and leachate from the landfills is primarily discharged into municipal sewers, with few exceptions. Previous research^61^ on 18 landfill sites identified several frequently detected PFAS in landfill leachates, including PFCAs (C4–C10), PFSAs (C4–C8), 6:2 FTCA, 8:2 FTCA, 5:3 FTCA, 7:3 FTCA, 6:2 FTS, 8:2 FTS, and FOSAAs. Another study^62^ also reported the frequent presence of PFCAs, PFSAs, FTSs, 6:2 diPAP, and *N*-MeFOSAA in landfill leachates. Landfills could contribute to elevated PFAS concentrations in specific situations, especially considering that they may discharge into smaller WWTPs, as they are typically situated outside city centers.^44^ Airports and fire training sites can also be a significant PFAS source due to the use of aqueous film forming foams (AFFFs). Previous research^39^ demonstrated that airports and fire training sites using AFFFs could contribute to increased levels of PFAS in municipal wastewater. AFFFs exhibit varying PFAS compositions depending on the manufacturers, but generally show high concentrations of 6:2 fluorotelomers (e.g., 6:2 FTS, 6:2 fluorotelomer sulfonamides),^63^ eventually transforming into PFAAs. Based on the considerations mentioned above, there is a potential impact from industrial PFAS point sources near WWTPs on the elevated concentrations of PFAS in municipal wastewater.

### 3.2 Fate of PFAAs Across WWTP Treatment Processes

To investigate the fate of PFAS within WWTPs, PFAS concentrations were compared across three wastewater samples: influents (INF), activated sludge effluents (AS EFF), and final effluents (EFF). SI Figure S2 shows the concentrations of PFAS in wastewater samples from nine WWTPs.

**Figure 2:**
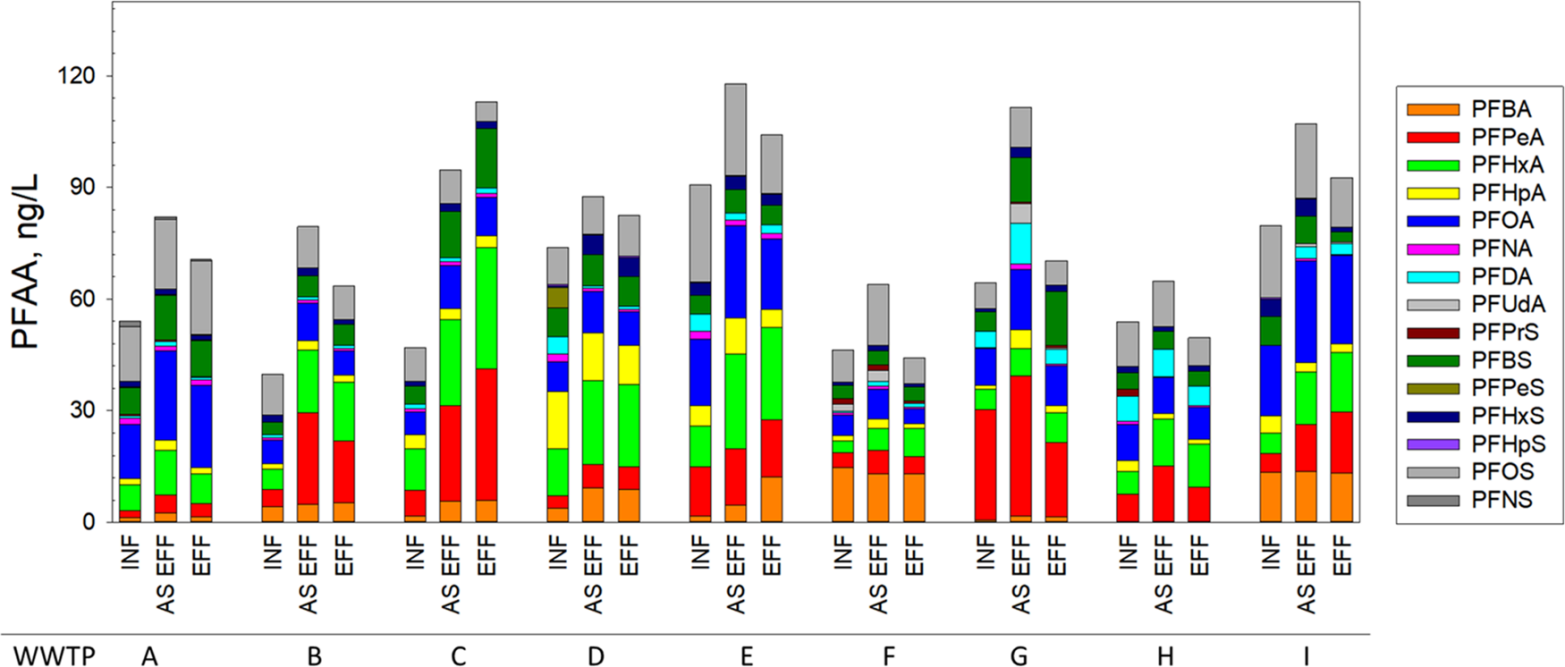
Concentrations of PFAAs in influents (INF), activated sludge effluent (AS EFF), and final effluent (EFF) in nine WWTPs (A–I).

∑PFCAs in AS EFF ranged from 40.9 (WWTP F) to 85.8 ng·L^–1^ (WWTP G) (SI Tables S3–S12 and Figure 2). Compared to INF, ∑PFCAs increased after activated sludge treatment. The increase of ∑PFCA after activated sludge treatment (expressed as Δ) ranged from 9.1 (WWTP F) to 39.5 ng·L^–1^ (WWTP C). The highest increase was observed for PFHxA (median Δ:8.7 ng·L^–1^) and PFPeA (median Δ:7.2 ng·L^–1^). The increase of PFCAs followed the trend: PFHxA > PFPeA > PFOA > PFBA > PFHpA ≫ others. The significant increase in short-chain PFHxA and PFPeA corresponds well with our previous research.^19^ Elevated concentrations of PFCAs following activated sludge treatment are likely attributable to the potential biotransformation of PFCA precursors into PFCAs. As previously stated, dominant known precursors in INF samples were 6:2 FTS, 6:2 diPAP, and 5:3 FTCA. 6:2 diPAP undergoes biotransformation, leading to the formation of 6:2 FTOH and 5:3 FTCA, which further metabolizes into PFHpA, PFHxA, and PFPeA over time.^49^ 6:2 FTS is biotransformed into 6:2 FTUCA, which further converts to PFHxA and PFPeA.^64^ ∑PFSAs in AS EFF ranged from 18.1 (WWTP H) to 45.9 ng·L^–1^ (WWTP G). Unlike PFCAs, the increase of PFSAs after activated sludge treatment was not significant, except for WWTP A (Δ:8.1 ng·L^–1^), WWTP C (Δ:8.3 ng·L^–1^), WWTP F (Δ:8.7 ng·L^–1^), and WWTP G (Δ:32.7 ng·L^–1^). The highest increase was observed for PFBS (median Δ:1.4 ng·L^–1^) and PFOS (median Δ:0.4 ng·L^–1^). PFSA precursors are sulfonamides, sulfoamidoethanols, and sulfoamidoacetic acids,^65,66^ including *N*-EtFOSAA, *N*-MeFOSAA, FBSA, and FOSA, which were detected in INF samples. PFOS is likely formed from the transformation of *N*-EtFOSAA, *N*-MeFOSAA, and FOSA, while PFBS is likely formed from FBSA.

Following activated sludge treatment, further advanced treatment processes employed at WWTPs (WWTP B: membrane bioreactor, UV; WWTP C: granular media filter, activated carbon filter, ozonation; WWTP D: UV; WWTP E: granular media filter, chlorination; WWTP F–I: UV) were found to be ineffective in reducing PFAA levels. The concentrations of PFAAs in final effluents (EFF) were similar to those in INF samples (Figure 2). These results align with previous studies, highlighting minimal PFAS removal during WWTP treatments.^15,19,67,68^ This demonstrates that municipal WWTPs can be significant point sources of PFAS contamination in receiving water and soil systems through either direct discharge or the application of recycled wastewater.^18,19,39^

In addition, PFAS removal efficiency (∑PFAS_INF_ – ∑PFAS_EFF_)/(∑PFAS_INF_) was compared based on the permitted treatment capacity (i.e., MGD), showing a no correlation (*R*^2^ = 0.09; SI Figure S3). This indicates that increasing treatment capacity does not necessarily lead to higher PFAS removal efficiency.

### 3.3 PFAA Precursors Estimated by TOP Assay

**Figure 3:**
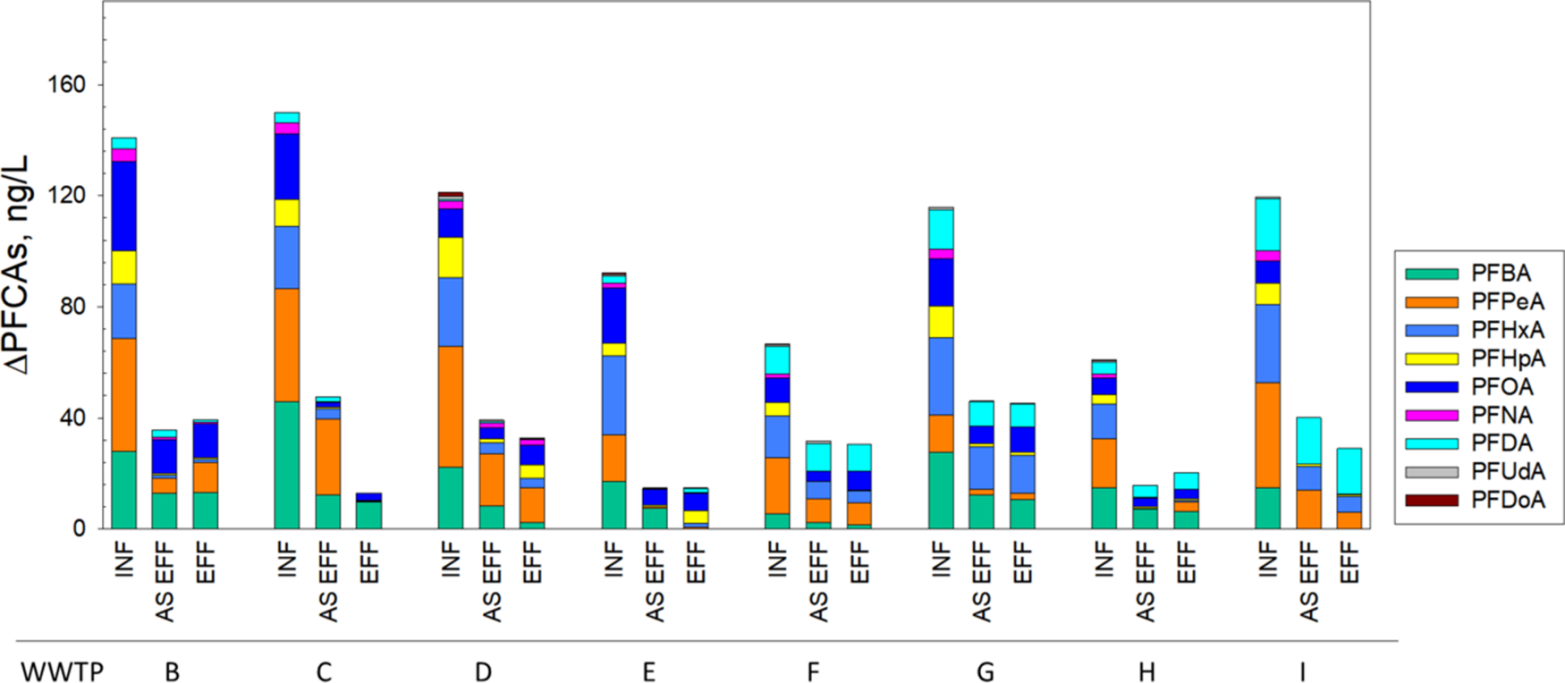
Increase of PFCA concentrations after TOP oxidation (ΔTOP) in influents (INF), activated sludge effluent (AS EFF), and final effluent (EFF) in eight WWTPs (B–I).

PFAA precursors can be indirectly estimated by the TOP assay. The TOP oxidation was conducted for wastewater samples from eight WWTPs (WWTP B-WWTP I). The TOP oxidation resulted in a 1.1- to 5.4-fold increase in PFCAs in wastewater samples. This finding aligns with observations from previous studies conducted on wastewater in the U.S., which reported PFCA increases of 3-fold^69^ and 1- to 8-fold.^67^Figure 3 and SI Tables S13–S20 present the concentrations of PFCAs following TOP oxidation, along with the concentration increase in PFCAs compared to prior to TOP oxidation (expressed as ΔTOP). Concentrations of PFCAs in INF after TOP oxidation ranged from 84.8 (WWTP F) to 181.6 ng·L^–1^ (WWTP C). Concentrations of PFCAs in INF samples increased after TOP oxidation compared to prior to TOP oxidation. The Δ_TOP_ ranged from 60.2 (WWTP H) to 149.9 ng·L^–1^ (WWTP C). The increase after TOP oxidation was greater for short-chain PFCAs than long-chain PFCAs in INF (ΔTOP of Σshort-chain PFCAs: 46.6 (WWTP F)–118.7 ng·L^–1^ (WWTP C); ΔTOP of Σlong-chain PFCAs: 11.9 (WWTP I)–50.8 ng·L^–1^ (WWTP G)). In particular, short-chain PFPeA and PFHxA exhibited the highest increase after TOP oxidation (median ΔTOP: 43.1 and 33.4 ng·L^–1^, respectively). This suggests that precursors in municipal wastewaters could be primarily transformed to short-chain PFCAs, rather than long-chain PFCAs. As stated above, INF samples include 6:2 FTS, 6:2 diPAP, and 5:3 FTCA with highest abundance. 6:2 FTS, 6:2 diPAP, and 5:3 FTCA are primarily transformed to short-chain PFCAs, such as PFBA, PFPeA, and PFHxA.^38,70^ In addition, municipal wastewater may very likely contain numerous unknown precursors that can be converted to short-chain PFCAs, as constantly observed in previous research.^46^ The increase after TOP oxidation substantially decreased after the activated sludge treatment (Figure 3), indicating that biological treatments have the potential to convert precursors to PFCAs, consequently reducing the ΔTOP of PFCAs. In addition, a strong correlation (*R*^2^ = 0.75, *p* < 0.01) between the increase in ∑short-chain PFCAs (expressed as Δ_ASEFF_) and the decrease in ΔTOP of short-chain PFCAs (ΔTOP_ASEFF_) after activated sludge treatment was observed (SI Figure S4). This suggests that some oxidizable short-chain precursors are likely transformed to short-chain PFCAs during activated sludge treatment. While WWTP B, WWTP C, and WWTP E employ further advanced treatment processes following activated sludge treatment, only WWTP C showed a significant decrease of ΔTOP of PFCAs (particularly short-chain PFCAs) through further advanced treatment processes. Note that WWTP C employs granular media filter, activated carbon filter and ozonation following the activated sludge treatment (Table 1).

### 3.4 PFAS Load Discharged from WWTPs

The maximum PFAS loads from WWTPs were estimated by multiplying the concentrations of PFAS detected in the final effluents (∑PFAS_EFF_) by the permitted MGD flow rate. The PFAS loads from WWTPs ranged from 340 (WWTP G) g·year^–1^ to 9645 g·year^–1^ (WWTP C). Five out of six PFAS (PFOA, PFOS, PFNA, PFBS, PFHxS, HFPO–DA), which are regulated for NPDWR permits, were detected in all final effluent samples, except for HFPO–DA. Among the six PFAS, PFOA and PFOS exhibited the highest loads, with median values of 295 and 276 g·year^–1^, respectively, in the effluents.

The NPDWR establishes standards for public drinking water quality in the U.S., setting MCLs for specific PFAS compounds: PFOA (4.0 ng·L^–1^), PFOS (4.0 ng·L^–1^), PFHxS (10 ng·L^–1^), PFNA (10 ng·L^–1^), and HFPO–DA (10 ng·L^–1^) as contaminants with individual MCLs. Additionally, for PFAS mixtures containing at least two or more of PFHxS, PFNA, HFPO–DA, and PFBS, a hazard index (HI) MCL is applied, with an HI value of 1. Although WWTP discharge is not directly used for drinking, it can eventually reach water sources used for drinking water after flowing through rivers and streams. Wastewater effluent may also be subject to active direct or indirect potable reuse. This study found that PFOA and PFOS were present in all WWTP effluents at concentrations exceeding 4 ng·L^–1^. Furthermore, the HI was calculated for the mixture containing two or more of four PFAS (PFNA, PFBS, PFHxS, and HFPO–DA) by summing their individual Hazard Quotients (HQs).
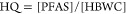
2
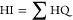
3where [PFAS] is measured concentration in effluent samples and [HBWC] is health-based water concentration, reported value by U.S. EPA. [HBWC] for PFNA, PFBS, PFHxS, and HFPO–DA is 10, 2000, 10, and 10 ng·L^–1^, respectively. The estimated HI in nine WWTP effluents ranged from 0.2 to 6.1, with a mean value of 1.9 and a median value of 0.6. Note that HI > 1.0 represents high hazard.

## 4 Conclusions

PFAS are emerging contaminants found in everyday products, leading to their release into wastewater. This study investigated PFAS levels in municipal wastewater from nine WWTPs, each with varying treatment capacity and processes. Municipal wastewaters contained high concentrations of short-chain PFCAs, PFOS, 5:3 FTCA, 6:2 FTS, and 6:2 diPAP, with maximum PFAS loads ranging from 378 to 9271 g·year^–1^ prior to treatment. Despite the phase out of PFOS, our findings revealed persistently high concentrations of PFOS in wastewater. Concentrations of PFCAs were found to be elevated after activated sludge treatment, especially for PFPeA and PFHxA, reflecting the transformation of precursors into PFCAs through biological reactions. Further advanced treatments showed limited effectiveness in removing PFAS. The TOP oxidation increased PFCA concentrations up to 5.4 times, with PFPeA and PFHxA exhibiting the highest increases. These findings suggest a shift toward the use of shorter-chain PFAS. The maximum possible PFAS loads discharged from WWTPs ranged from 340 to 9645 g·year^–1^, similar to PFAS loads entering the WWTPs. PFOA and PFOS were detected in all WWTP effluents at concentrations exceeding 4 ng·L^–1^, and HI for mixtures containing 2 or more of 4 PFAS (PFNA, PFBS, PFHxS, and HFPO–DA) was 0.2–6.1. While an HI exceeding 1.0 indicates a potential health concern for drinking water, further research is needed to understand the specific risks implicated by PFAS in wastewaters. These results suggest that wastewater discharges may pose a potential risk and highlight the need for improved PFAS removal strategies in wastewater treatment.

## Supporting Information Available

PFAS chemicals, analytical standards, isotope-labeled surrogates, other chemicals, and analytical quality control (Texts S1–S2); 40 PFAS analyzed in this study (Table S1); PFAS analytes, retention time, and isotope-labeled surrogates (Table S2); quality control sample results for PFAS analytes (Table S3); PFAS concentrations (ng·L^–1^) in wastewater samples (Tables S4–S12); PFCA concentrations (ng·L^–1^) in wastewater samples after TOP oxidation (Tables S13–S20); relationship between summed PFSAs and PFCAs in municipal wastewater (Figure S1); concentrations of short-chain PFAAs, long-chain PFAAs, precursors, and other PFAS in influents (INF), biological reactor basin effluent (BRB), and final effluent (EFF) (Figure S2); relationship between MGD of WWTPs and PFAS removal efficiency (∑PFAS_INF_ – ∑PFAS_EFF_) (Figure S3) (PDF)
