# Supplementary material for: Occurrence, Fate, and Removal of Per- and Polyfluoroalkyl Substances (PFAS) in Small- and Large-Scale Municipal Wastewater Treatment Facilities in the United States
Source: ACS ES T Water. 2024 Nov 15;4(12):5428–36. doi: 10.1021/acsestwater.4c00541 (PMC11650586; doi:10.1021/acsestwater.4c00541)
Supplement: Supplementary file 1 — ew4c00541_si_001.pdf [file ew4c00541_si_001.pdf]

Supporting Information:

Occurrence, Fate and Removal of Per- and Polyfluoroalkyl Substances (PFAS) in Small- and Large-Scale Municipal Wastewater Treatment Facilities in the United States

Juhee Kim,[a,c] Xiaoyue Xin,[a] Gary L. Hawkins,[b] Qingguo Huang,[b] and Ching-Hua Huang[a,*]

[a]School of Civil and Environmental Engineering, Georgia Institute of Technology, Atlanta, GA 30332, United States

[b]Department of Crop and Soil Sciences, University of Georgia, Griffin, GA 30223, United States

[c]Department of Civil, Environmental and Construction Engineering, University of Hawai`i at Mānoa, Honolulu, HI 96822, United States

[*]Corresponding Author. Emails: ching-hua.huang@ce.gatech.edu (Ching-Hua Huang)

Supporting information contains:

- 48 Pages
- 2 Texts
- 20 Tables
- 4 Figures

Contents

Texts

Text S1. PFAS chemicals, analytical standards, isotope-labeled surrogates, and other chemicals. ---------S3

Text S2. Analytical Quality Control (QC). --S5

Tables

Table S1. The 40 PFAS analyzed in this study.--S6

Table S2. PFAS analytes, retention time, and isotope-labeled surrogates.-------------------------------------S7

Table S3. Quality control sample results for PFAS analytes.--S8

Table S4-S12. PFAS concentrations (ng·L^{-1}) in wastewater samples in WWTP A-WWTP I.-------- S9-S35

Table S13-S20. PFCA concentrations (ng·L^{-1}) in wastewater samples in WWTP B after TOP oxidation.--- --- S36-S43

Figures

Figure S1. Relationship between summed PFSAs and PFCAs in municipal wastewater entering into 9 WWTPs.---S44

Figure S2. Concentrations of short-chain PFAAs (PFCAs < C8, PFSAs < C6), long-chain PFAAs, precursors, and other PFAS in influents (INF), biological reactor basin effluent (BRB), and final effluent (EFF) in 9 WWTPs.---S45

Figure S3. Relationship between MGD of WWTPs and PFAS removal efficiency ($\sum PFAS_{INF}$-$\sum PFAS_{EFF}$). ---S46

Figure S4. Relationship between the increase of ∑short-chain PFCAs after activated sludge treatment (expressed as Δ_{ASEFF}) and the decrease of ΔTOP of short-chain PFCAs after activated sludge treatment (expressed as ΔTOP_{ASEFF}). ---S47

Text S1. PFAS chemicals, analytical standards, isotope-labeled surrogates, and other chemicals

The 40 PFAS were purchased from Wellington Laboratories (Guelph, Canada): perfluorobutanoic acid (PFBA), perfluoropentanoic acid (PFPeA), perfluorohexanoic acid (PFHxA), perfluoroheptanoic acid (PFHpA), perfluorooctanoic acid (PFOA), perfluorononanoic acid (PFNA), perfluorodecanoic acid (PFDA), perfluoroundecanoic acid (PFUnA), perfluorododecanoic acid (PFDoA), perfluorortridecanoic acid (PFTrDA), perfluorotetradecanoic acid (PFTeDA), perfluoropropane sulfonic acid (PFPrS), perfluorobutane sulfonic acid (PFBS), perfluoropentane sulfonic acid (PFPS), perfluorohexane sulfonic acid (PFHxS), perfluorheptane sulfonic acid (PFHpS), perfluorooctane sulfonic acid (PFOS), perfluorononane sulfonic acid (PFNS), perfluorodecane sulfonic acid (PFDS), 4:2 fluorotelomer sulfonic acid (4:2 FTS), 6:2 fluorotelomer sulfonic acid (6:2 FTS), 8:2 fluorotelomer sulfonic acid (8:2 FTS), 6:2 fluorotelomer carboxylic acid (6:2 FTCA), 5:3 fluorotelomer carboxylic acid (5:3 FTCA), 6:2 fluorotelomer unsaturated carboxylic acid (6:2 FTUCA), perfluorobutane sulfonamide (FBSA), perfluorohexane sulfonamide (FHxSA), perfluorooctane sulfonamide (PFOSA), N-methyl-perfluoro-1-octanesulfonamidoacetic acid (N-MeFOSAA), N-ethyl-perfluoro-1-octanesulfonamidoacetic acid (N-EtFOSAA), hexafluoropropylene oxide-dimer acid (HFPO-DA), 4,8-dioxa-3H-perfluorononanoic acid (ADONA), 9-chlorohexadecafluoro-3-oxanone-1-sulfonic acid (9Cl-PF3ONS), 11-chloroeicosafluoro-3-oxaundecane-1-sulfonic acid (11Cl-PF3OUdS), perfluoro(2-ethoxyethane)sulfonic acid (PFEESA), perfluoro-3-methoxypropanoic acid (PF4OPeA), perfluoro-4-methoxybutanoic acid (PF5OHxA), nonafluoro-3,6-dioxaheptanoic acid (3,6-OPFHpA), 6:2 fluorotelomer phosphate diester (6:2 diPAP), and 6:2/8:2 fluorotelomer phosphate diester (6:2/8:2 diPAP).

Twenty-three isotope-labeled surrogates were purchased from Wellington Laboratories (Guelph, ON): [$^{13}C_4$] PFBA, [$^{13}C_5$] PFPeA, [$^{13}C_5$] PFHxA, [$^{13}C_4$] PFHpA, [$^{13}C_8$] PFOA, [$^{13}C_9$] PFNA, [$^{13}C_6$] PFDA, [$^{13}C_7$] PFUnA, [$^{13}C_2$] PFDoA, [$^{13}C_2$] PFTeA, [$^{13}C_3$] PFBS, [$^{13}C_5$] PFHxS, [$^{13}C_8$] PFOS, [$^{13}C_2$] 4:2 FTS, [$^{13}C_2$] 6:2 FTS, [$^{13}C_2$] 8:2 FTS, [$^{13}C_2$] 6:2 FTCA, [$^{13}C_2$] 6:2 FTUCA, [$^{13}C_8$] PFOSA, [$^{2}D_3$] N-MeFOSAA, [$^{2}D_5$] N-EtFOSAA, [$^{13}C_3$] HFPO-DA, and [$^{13}C_2$] 6:2 diPAP.

Sodium phosphate dibasic (≥99%; MilliporeSigma), sodium phosphate monobasic (≥99.0%; MilliporeSigma), sodium hydroxide (97%; Thermo Fisher Scientific), ammonium hydroxide (30% NH_3 basis, MilliporeSigma), ammonium acetate (7.5 M solution; MilliporeSigma), and acetic acid (≥99%; MilliporeSigma) were used as solid-phase extraction (SPE) reagents. LC-MS grade methanol (Fisher Optima), LC-MS grade acetonitrile (Fisher Optima), acetic acid (Fisher Optima), LC-MS grade ammonium acetate (Fisher Optima), and Milli-Q water were used to prepare LC eluent.

Text S2. Analytical Quality Control (QC)

The data censoring and qualification followed our previous study:[1]

1. Identification

a. Extracted-ion chromatogram (EIC): EIC was extracted with m/z expansion of ± 10 ppm.

b. Retention time: retention time of the analyte should match that of the standard with a tolerance of ± 0.15 min.

c. Mass error: m/z of the analyte should match the theoretical m/z with a tolerance of ± 5 ppm.

d. Isotope pattern: isotope patterns of the analyte should match the theoretical isotope patterns with deviation of $<5\%$.

2. Quantification

a. LOD: Concentration should be greater than LOD which was defined as "3×SNR" (signal to noise ratio ≥ 3) and indicated in Table S3.

b. Extraction blank: Concentration in sample should be greater than the MDL (method detection limit). Method detection limits (MDLs) were determined by method extraction blanks and the results shown in Table S3. Method blanks (n=7) consisted of 500 mL of Milli-Q DI water without analyte spikes. Sample MDLs for each analyte were selected as the maximum observed method blank concentration. All reported concentrations of the analytes should be greater than the corresponding MDLs found in extraction blanks.

Table S1. The 40 PFAS analyzed in this study.

Acronym	Chemical Name	Chemical Formula
Perfluorocarboxylic Acids		
PFBA	Perfluorobutanoic acid	$C_3F_7CO_2H$
PFPeA	Perfluoropentanoic acid	$C_4F_9CO_2H$
PFHxA	Perfluorohexanoic acid	$C_5F_{11}CO_2H$
PFHpA	Perfluoroheptanoic acid	$C_6F_{13}CO_2H$
PFOA	Perfluorooctanoic acid	$C_7F_{15}CO_2H$
PFNA	Perfluorononanoic acid	$C_8F_{17}CO_2H$
PFDA	Perfluorodecanoic acid	$C_9F_{19}CO_2H$
PFUnA	Perfluoroundecanoic acid	$C_{10}F_{21}CO_2H$
PFDoA	Perfluorododecanoic acid	$C_{11}F_{23}CO_2H$
PFTrDA	Perfluorortridecanoic acid	$C_{12}F_{25}CO_2H$
PFTeDA	Perfluorotetradecanoic acid	$C_{13}F_{27}CO_2H$
Perfluorosulfonic Acids		
PFPrS	Perfluoropropane sulfonic acid	$C_3F_7SO_3H$
PFBS	Perfluorobutane sulfonic acid	$C_4F_9SO_3H$
PFPeS	Perfluoropentane sulfonic acid	$C_5F_{11}SO_3H$
PFHxS	Perfluorohexane sulfonic acid	$C_6F_{13}SO_3H$
PFHpS	Perfluorheptane sulfonic acid	$C_7F_{15}SO_3H$
PFOS	Perfluorooctane sulfonic acid	$C_8F_{17}SO_3H$
PFNS	Perfluorononane sulfonic acid	$C_9F_{19}SO_3H$
PFDS	Perfluorodecane sulfonic acid	$C_{10}F_{21}SO_3H$
Fluorotelomer Sulfonic Acids		
4:2 FTS	4:2 Fluorotelomer sulfonic acid	$C_6H_4F_9SO_3H$
6:2 FTS	6:2 Fluorotelomer sulfonic acid	$C_8H_4F_{13}SO_3H$
8:2 FTS	8:2 Fluorotelomer sulfonic acid	$C_{10}H_4F_{17}SO_3H$
Fluorotelomer Carboxylic Acids		
6:2 FTCA	6:2 Fluorotelomer carboxylic acid	$C_6F_{13}CH_2CO_2H$
5:3 FTCA	5:3 Fluorotelomer carboxylic acid	$C_5CH_2CH_2F_{11}CO_2H$
Fluorotelomer Unsaturated Carboxylic Acids		
6:2 FTUCA	6:2 Fluorotelomer unsaturated carboxylic acid	$C_6F_{12}CHCO_2H$
Perfluorosulfonamides		
FBSA	Perfluorobutane sulfonamide	$C_4F_9SO_2NH_2$
FHxSA	Perfluorohexane sulfonamide	$C_6F_{13}SO_2NH_2$
PFOSA	Perfluorooctane sulfonamide	$C_8F_{17}SO_2NH_2$
Perfluorosulfonamidoacetic Acids		
N-MeFOSAA	N-Methyl-perfluoro-1-octanesulfonamidoacetic acid	$C_8F_{17}SO_2NHC_3O_2H_5$
N-EtFOSAA	N-Ethyl-perfluoro-1-octanesulfonamidoacetic acid	$C_8F_{17}SO_2NHC_4O_2H_7$
Per- and Polyfluoroethers		
HFPO-DA	Hexafluoropropylene oxide-dimer acid	$C_3F_7OC_2F_4CO_2H$
ADONA	4,8-dioxa-3H-perfluorononanoic acid	$CF_3OC_3F_6OC_2F_3HCO_2H$
9Cl-PF3ONS	9-chlorohexadecafluoro-3-oxanone-1-sulfonic acid	$ClC_6F_{12}OC_2F_4SO_3H$
11Cl-PF3OUdS	11-chloroeicosafluoro-3-oxaundecane-1-sulfonic acid	$ClC_8F_{16}OC_2F_4SO_3H$
PFEESA	Perfluoro(2-ethoxyethane)sulfonic acid	$C_2F_5OC_2F_4SO_3H$
PF4OPeA	Perfluoro-3-methoxypropanoic acid	$CF_3OC_2F_4CO_2H$
PF5OHxA	Perfluoro-4-methoxybutanoic acid	$CF_3OC_3F_6CO_2H$
3,6-OPFHpA	Nonafluoro-3,6-dioxaheptanoic acid	$CF_3OC_2F_4OCF_2CO_2H$
Fluorotelomer Phosphate Diesters		
6:2 diPAP	6:2 Fluorotelomer phosphate diester	$C_6F_{13}C_2H_4PO_4HC_2H_4C_6F_{13}$
6:2/8:2 diPAP	6:2/8:2 Fluorotelomer phosphate diester	$C_6F_{13}C_2H_4PO_4HC_2H_4C_8F_{17}$

Table S2. PFAS analytes, retention time, and isotope-labeled surrogates.

No.	Analyte	Theoretical m/z	Retention Time (min)	Isotope-labeled Surrogate	Theoretical m/z
1	PFBA	212.9792	7.5	[$^{13}C_4$] PFBA	216.9926
2	PFPeA	262.976	8.6	[$^{13}C_5$] PFPeA	267.9928
3	PFHxA	312.9728	9.3	[$^{13}C_5$] PFHxA	317.9896
4	PFHpA	362.9696	10.2	[$^{13}C_4$] PFHpA	366.983
5	PFOA	412.9664	11.1	[$^{13}C_8$] PFOA	420.9933
6	PFNA	462.9632	12.1	[$^{13}C_9$] PFNA	471.9934
7	PFDA	512.9600	13.0	[$^{13}C_6$] PFDA	518.9802
8	PFUnA	562.9568	13.8	[$^{13}C_7$] PFUnA	569.9803
9	PFDoA	612.9537	14.6	[$^{13}C_2$] PFDoA	614.9604
10	PFTrDA	662.9505	15.3	[$^{13}C_2$] PFTeA	714.954
11	PFTeDA	712.9473	15.8	[$^{13}C_2$] PFTeA	714.954
12	PFPrS	248.9462	8.1	[$^{13}C_3$] PFBS	301.9531
13	PFBS	298.9430	8.8	[$^{13}C_3$] PFBS	301.9531
14	PFPeS	348.9398	9.5	[$^{13}C_5$] PFHxS	401.9467
15	PFHxS	398.9366	10.4	[$^{13}C_3$] PFHxS	401.9467
16	PFHpS	448.9334	11.3	[$^{13}C_8$] PFOS	506.9571
17	PFOS	498.9302	12.2	[$^{13}C_8$] PFOS	506.9571
18	PFNS	548.9270	13.1	[$^{13}C_6$] PFDA	518.9802
19	PFDS	598.9238	13.9	[$^{13}C_7$] PFUnA	569.9803
20	4:2 FTS	326.9743	9.2	[$^{13}C_2$] 4:2 FTS	328.981
21	6:2 FTS	426.9679	10.9	[$^{13}C_2$] 6:2 FTS	428.9746
22	8:2 FTS	526.9679	12.9	[$^{13}C_2$] 8:2 FTS	528.9682
23	6:2FTCA	376.9853	10.3	[$^{13}C_2$] 6:2 FTCA	378.992
24	5:3FTCA	341.0041	10.2	[$^{13}C_2$] 6:2 FTCA	378.992
25	6:2 FTUCA	356.9790	10.3	[$^{13}C_2$] 6:2 FTUCA	358.9858
26	FBSA	297.9590	9.6	[$^{13}C_3$] PFHxS	401.9467
27	FHxSA	397.9526	11.8	[$^{13}C_8$] PFOS	506.9571
28	PFOSA	497.9462	13.9	[$^{13}C_8$] PFOSA	505.973
29	N-MeFOSAA	569.9673	13.3	[$^{2}D_3$] N-MeFOSAA	572.9862
30	N-EtFOSAA	583.9830	13.8	[$^{2}D_5$] N-EtFOSAA	589.0144
31	HFPO-DA	284.9779	9.6	[$^{13}C_3$] HFPO-DA	286.9846
32	ADONA	376.9689	10.3	[$^{13}C_3$] PFHpA	366.983
33	9Cl-PF3ONS	530.8956	13	[$^{13}C_8$] PFOS	506.9571
34	11Cl-PF3OUdS	630.8892	14.5	[$^{13}C_7$] PFUnA	569.9803
35	PFEESA	314.9379	9.5	[$^{13}C_3$] PFBS	301.9531
36	PF4OPeA	228.9741	8.4	[$^{13}C_4$] PFBA	216.9926
37	PF5OHxA	278.9709	9.2	[$^{13}C_5$] PFPeA	267.9928
38	3,6-OPFHpA	200.9792	9.6	[$^{13}C_5$] PFHxA	317.9896
39	6:2 diPAP	788.9751	15.6	[$^{13}C_2$] 6:2 diPAP	792.9885
40	6:2/8:2 diPAP	888.9687	16.4	[$^{13}C_2$] 6:2 diPAP	792.9885

Table S3. Quality control sample results for PFAS analytes.

No.	Analyte	Extraction Blanks (n=7)		DI Water Spikes (10.0 ng/L, n=7)		LOD[c] (ng/L)
		n Detects	Max (ng/L)	Mean %R[a]	%RSD[b]	
1	PFBA	6	0.27	103.7	3.0	0.1
2	PFPeA	7	0.22	97.4	4.2	0.1
3	PFHxA	6	0.31	92.8	6.6	0.1
4	PFHpA	5	0.53	96.5	2.9	0.1
5	PFOA	7	0.72	104.4	5.3	0.1
6	PFNA	0	0	94.8	1.3	0.1
7	PFDA	0	0	102.5	3.7	0.1
8	PFUnA	0	0	96.3	5.3	0.1
9	PFDoA	0	0	88.6	6.2	0.1
10	PFTrDA	0	0	83.9	5.4	0.1
11	PFTeDA	0	0	90.2	4.4	0.1
12	PFPrS	0	0	82.5 (n=5)	6.2	0.2
13	PFBS	1	< LOD	97.8	3.0	0.1
14	PFPeS	0	0	96.2	3.0	0.1
15	PFHxS	3	0.13	96.9	2.1	0.1
16	PFHpS	0	0	106.7	6.4	0.1
17	PFOS	7	0.96	101.5	5.2	0.1
18	PFNS	0	0	83.3	5.3	0.1
19	PFDS	0	0	93.1	7.0	0.1
20	6:2 FTCA	7	0.34	124.3 (n=5)	6.8	0.2
21	6:2 FTCUA	1	< LOD	131.3 (n=5)	7.7	0.2
22	5:3 FTCA	7	1.30	122.2 (n=5)	9.2	0.2
23	4:2 FTS	2	< LOD	97.6 (40 ng/L)	3.5	0.2
24	6:2 FTS	7	1.53	137.4 (40 ng/L)	11.2	0.2
25	8:2 FTS	2	< LOD	99.5 (40 ng/L)	2.3	0.2
26	6:2 diPAP	1	< LOD	119.9 (n=5)	6.6	0.2
27	6:2/8:2 diPAP	0	0	126.6 (n=5)	8.4	0.2
28	PFOSA	4	0.19	102.8	5.5	0.2
29	NMeFOSAA	0	0	95.8	9.9	0.2
30	NEtFOSAA	0	0	98.0	4.8	0.2
31	FBSA	3	< LOD	33.8	5.5	0.2
32	FHxSA	1	< LOD	75.4	9.9	0.2
33	9Cl-PF3ONS	0	0	87.5	3.6	0.2
34	11Cl-PF3OUdS	0	0	84.0	7.5	0.2
35	HFPO-DA	5	0.27	96.6	2.4	0.2
36	ADONA	0	0	94.3	2.3	0.2
37	PFMPA	0	0	89.9	3.8	0.2
38	PFMBA	0	0	103.0	8.4	0.2
39	PFEESA	0	0	92.7	3.7	0.2
40	NFDHA	0	0	98.1	3.0	0.2

[a] %R = percent recovery.
[b] %RSD = percent relative standard deviation.
[c] LOD = limit of detection

Table S4. PFAS concentrations (ng·L^{-1}) in wastewater samples in WWTP A (Sampling date: August 2023).

PFAS	ng/L	Primary INF	Activated Sludge EFF	Final EFF
PFBA	**Average**[a]	**1.3**	**2.6**	**1.4**
	STDEV[a]	0.0	0.5	0.0
PFPeA	**Average**	**1.9**	**4.8**	**3.6**
	STDEV	0.5	1.3	0.1
PFHxA	**Average**	**7.0**	**12.0**	**8.0**
	STDEV	1.5	0.3	0.0
PFHpA	**Average**	**1.5**	**2.7**	**1.7**
	STDEV	0.1	0.3	0.2
PFOA	**Average**	**14.6**	**23.9**	**22.1**
	STDEV	0.1	3.5	0.2
PFNA	**Average**	**1.6**	**1.4**	**1.5**
	STDEV	0.4	0.0	0.6
PFDA	**Average**	**0.8**	**1.1**	**0.5**
	STDEV	0.4	0.1	0.2
PFUdA	**Average**	**<LOD**[b]	**<LOD**	**<LOD**
	STDEV			
PFDoA	**Average**	**ND**[c]	**ND**	**ND**
	STDEV			
PFTrDA	**Average**	**ND**	**ND**	**ND**
	STDEV			
PFTeDA	**Average**	**ND**	**ND**	**ND**
	STDEV			
PFPrS	**Average**	**0.3**	**0.4**	**0.3**
	STDEV	0.1	0.2	0.1
PFBS	**Average**	**7.3**	**12.1**	**9.8**
	STDEV	0.1	3.7	0.1
PFPeS	**Average**	**0.2**	**0.2**	**0.2**
	STDEV	0.1	0.1	0.0
PFHxS	**Average**	**1.3**	**1.4**	**1.2**
	STDEV	0.0	0.3	0.0
PFHpS	**Average**	**<LOD**	**<LOD**	**<LOD**
	STDEV			
PFOS	**Average**	**14.9**	**18.8**	**19.8**
	STDEV	1.4	1.6	1.8
PFNS	**Average**	**1.4**	**0.6**	**0.4**
	STDEV	0.5	0.1	0.2

PFDS	**Average**	**<LOD**	**<LOD**	**<LOD**
	STDEV			
6:2 FTCA	**Average**	**ND**	**ND**	**ND**
	STDEV			
6:2 FTUCA	**Average**	**ND**	**ND**	**ND**
	STDEV			
5:3 FTCA	**Average**	**ND**	**ND**	**ND**
	STDEV			
4:2 FTS	**Average**	**ND**	**ND**	**ND**
	STDEV			
6:2 FTS	**Average**	**1.7**	**2.3**	**1.7**
	STDEV	0.6	0.2	0.3
8:2 FTS	**Average**	**ND**	**ND**	**ND**
	STDEV			
6:2 diPAP	**Average**	**ND**	**ND**	**ND**
	STDEV			
6:2/8:2 diPAP	**Average**	**ND**	**ND**	**ND**
	STDEV			
FOSA	**Average**	**<LOD**	**<LOD**	**<LOD**
	STDEV			
nMeFOSAA	**Average**	**18.0**	**11.5**	**11.5**
	STDEV	7.5	0.6	0.6
nEtFOSAA	**Average**	**ND**	**ND**	**ND**
	STDEV			
FBSA	**Average**	**<LOD**	**<LOD**	**<LOD**
	STDEV			
FHxSA	**Average**	**<LOD**	**<LOD**	**<LOD**
	STDEV			
HFPO-DA	**Average**	**19.4**	**29.9**	**29.6**
	STDEV	1.3	6.9	1.1
NaDONA	**Average**	**ND**	**ND**	**ND**
	STDEV			
PFMPA	**Average**	**ND**	**ND**	**ND**
	STDEV			
PFBMA	**Average**	**ND**	**ND**	**ND**
	STDEV			
NFDHA	**Average**	**ND**	**ND**	**ND**
	STDEV			
PFEESA	**Average**	**ND**	**ND**	**ND**
	STDEV			

PF3ONS	**Average**	**ND**	**ND**	**ND**
	STDEV			
PF3OUdS	**Average**	**ND**	**ND**	**ND**
	STDEV			
**$\sum_{11}$PFCAs**		**28.7**	**48.6**	**38.8**
**$\sum_{8}$PFSAs**		**25.4**	**33.5**	**31.8**
**$\sum_{19}$PFAAs**		**54.1**	**82.1**	**70.6**
**$\sum_{7}$Short-chain PFAAs**		**19.5**	**34.8**	**25.0**
**$\sum_{12}$Long-chain PFAAs**		**34.7**	**47.3**	**45.6**
**$\sum_{21}$Precursors**		**39.1**	**40.6**	**42.8**
**$\sum_{40}$PFAS**		**93.2**	**122.4**	**113.5**

[a] Average value and standard deviation for the duplicate. [b]Limit of detection [c]Not detected

Table S5. PFAS concentrations ($ng \cdot L^{-1}$) in wastewater samples in WWTP B (Sampling date: September 2021).

PFAS	ng/L	Primary INF	Activated Sludge EFF	Final EFF
PFBA	**Average**[a]	**4.2**	**4.8**	**5.3**
	STDEV[a]	0.2	1.4	0.9
PFPeA	**Average**	**4.7**	**24.6**	**16.6**
	STDEV	0.7	3.8	1.2
PFHxA	**Average**	**5.3**	**16.9**	**15.9**
	STDEV	0.7	0.8	0.2
PFHpA	**Average**	**1.6**	**2.4**	**1.8**
	STDEV	0.1	0.1	0.1
PFOA	**Average**	**6.2**	**10.2**	**6.6**
	STDEV	0.2	0.2	0.6
PFNA	**Average**	**0.7**	**0.7**	**0.5**
	STDEV	0.0	0.0	0.0
PFDA	**Average**	**0.7**	**0.9**	**1.0**
	STDEV	0.0	0.0	0.1
PFUdA	**Average**	**ND**[b]	**ND**	**ND**
	STDEV			
PFDoA	**Average**	**ND**	**ND**	**ND**
	STDEV			
PFTrDA	**Average**	**ND**	**ND**	**ND**
	STDEV			
PFTeDA	**Average**	**ND**	**ND**	**ND**
	STDEV			
PFPrS	**Average**	**ND**	**ND**	**ND**
	STDEV			
PFBS	**Average**	**3.5**	**5.7**	**5.5**
	STDEV	0.3	0.3	0.0
PFPeS	**Average**	**<LOD**[c]	**<LOD**	**<LOD**
	STDEV			
PFHxS	**Average**	**1.9**	**2.0**	**1.3**
	STDEV	0.1	0.2	0.1
PFHpS	**Average**	**<LOD**	**<LOD**	**<LOD**
	STDEV			
PFOS	**Average**	**10.9**	**11.1**	**9.1**
	STDEV	1.7	0.6	0.9
PFNS	**Average**	**ND**	**ND**	**ND**
	STDEV			

PFDS	**Average**	**ND**	**ND**	**ND**
	STDEV			
6:2 FTCA	**Average**	**2.7**	**<LOD**	**<LOD**
	STDEV	0.1		
6:2 FTUCA	**Average**	**5.6**	**<LOD**	**<LOD**
	STDEV	0.0		
5:3 FTCA	**Average**	**3.0**	**<LOD**	**<LOD**
	STDEV	0.4		
4:2 FTS	**Average**	**ND**	**ND**	**ND**
	STDEV			
6:2 FTS	**Average**	**2.7**	**<MDL[d]**	**0.8**
	STDEV	0.8		0.2
8:2 FTS	**Average**	**<LOD**	**<LOD**	**<LOD**
	STDEV			
6:2 diPAP	**Average**	**53.1**	**<LOD**	**<LOD**
	STDEV	0.0		
6:2/8:2 diPAP	**Average**	**2.3**	**<LOD**	**<LOD**
	STDEV	0.4		
FOSA	**Average**	**<LOD**	**<LOD**	**<LOD**
	STDEV			
nMeFOSAA	**Average**	**<LOD**	**<LOD**	**<LOD**
	STDEV			
nEtFOSAA	**Average**	**<LOD**	**<LOD**	**<LOD**
	STDEV			
FBSA	**Average**	**ND**	**ND**	**ND**
	STDEV			
FHxSA	**Average**	**<LOD**	**<LOD**	**<LOD**
	STDEV			
HFPO-DA	**Average**	**<LOD**	**<LOD**	**<LOD**
	STDEV			
NaDONA	**Average**	**ND**	**ND**	**ND**
	STDEV			
PFMPA	**Average**	**ND**	**ND**	**ND**
	STDEV			
PFBMA	**Average**	**ND**	**ND**	**ND**
	STDEV			
NFDHA	**Average**	**ND**	**ND**	**ND**
	STDEV			
PFEESA	**Average**	**ND**	**ND**	**ND**
	STDEV			

PF3ONS	**Average**	**ND**	**ND**	**ND**
	STDEV			
PF3OUdS	**Average**	**ND**	**ND**	**ND**
	STDEV			
**$\sum_{11}$PFCAs**		**23.5**	**60.6**	**47.7**
**$\sum_{8}$PFSAs**		**16.4**	**18.8**	**15.9**
**$\sum_{19}$PFAAs**		**39.9**	**79.5**	**63.6**
**$\sum_{7}$Short-chain PFAAs**		**19.3**	**54.4**	**45.1**
**$\sum_{12}$Long-chain PFAAs**		**20.6**	**25.0**	**18.5**
**$\sum_{21}$Precursors**		**69.5**	**0.4**	**0.8**
**$\sum_{40}$PFAS**		**109.3**	**79.9**	**64.4**

[a] Average value and standard deviation for the duplicate. [b]Not detected [c]Limit of detection [d]Method detection limit

Table S6. PFAS concentrations (ng·L^{-1}) in wastewater samples in WWTP C (Sampling date: September 2021).

PFAS	ng/L	Primary INF	Activated Sludge EFF	Final EFF
PFBA	**Average**[a]	**1.8**	**5.6**	**5.9**
	STDEV[a]	0.1	0.2	0.3
PFPeA	**Average**	**6.8**	**25.8**	**35.3**
	STDEV	0.2	4.7	0.1
PFHxA	**Average**	**11.2**	**23.1**	**32.6**
	STDEV	2.7	1.5	0.5
PFHpA	**Average**	**3.8**	**2.9**	**3.1**
	STDEV	0.1	0.3	0.0
PFOA	**Average**	**6.1**	**11.6**	**10.3**
	STDEV	0.2	0.9	0.4
PFNA	**Average**	**0.8**	**0.9**	**1.2**
	STDEV	0.0	0.1	0.0
PFDA	**Average**	**1.2**	**1.2**	**1.4**
	STDEV	0.0	0.0	0.2
PFUdA	**Average**	**<LOD**[b]	**<LOD**	**<LOD**
	STDEV			
PFDoA	**Average**	**ND**	**ND**	**ND**
	STDEV			
PFTrDA	**Average**	**ND**	**ND**	**ND**
	STDEV			
PFTeDA	**Average**	**ND**	**ND**	**ND**
	STDEV			
PFPrS	**Average**	**ND**	**ND**	**ND**
	STDEV			
PFBS	**Average**	**4.9**	**12.4**	**16.0**
	STDEV	0.2	0.2	0.1
PFPeS	**Average**	**<LOD**	**<LOD**	**<LOD**
	STDEV			
PFHxS	**Average**	**1.2**	**2.0**	**2.0**
	STDEV	0.1	0.1	0.1
PFHpS	**Average**	**<LOD**	**<LOD**	**<LOD**
	STDEV			
PFOS	**Average**	**9.1**	**9.1**	**5.2**
	STDEV	0.6	0.6	0.1
PFNS	**Average**	**ND**[c]	**ND**	**ND**
	STDEV			

PFDS	**Average**	**ND**	**ND**	**ND**
	STDEV			
6:2 FTCA	**Average**	**4.4**	**<LOD**	**<LOD**
	STDEV	0.1		
6:2 FTUCA	**Average**	**1.7**	**<LOD**	**<LOD**
	STDEV	0.5		
5:3 FTCA	**Average**	**19.9**	**<LOD**	**<LOD**
	STDEV	2.1		
4:2 FTS	**Average**	**ND**	**<LOD**	**<LOD**
	STDEV			
6:2 FTS	**Average**	**1.8**	**3.4**	**3.1**
	STDEV	0.3	0.3	0.2
8:2 FTS	**Average**	**<LOD**	**<LOD**	**<LOD**
	STDEV			
6:2 diPAP	**Average**	**19.9**	**<LOD**	**<LOD**
	STDEV	1.5		
6:2/8:2 diPAP	**Average**	**18.7**	**<LOD**	**<LOD**
	STDEV	0.9		
FOSA	**Average**	**ND**	**ND**	**ND**
	STDEV			
nMeFOSAA	**Average**	**ND**	**ND**	**ND**
	STDEV			
nEtFOSAA	**Average**	**ND**	**ND**	**ND**
	STDEV			
FBSA	**Average**	**ND**	**ND**	**ND**
	STDEV			
FHxSA	**Average**	**ND**	**ND**	**ND**
	STDEV			
HFPO-DA	**Average**	**ND**	**ND**	**ND**
	STDEV			
NaDONA	**Average**	**ND**	**ND**	**ND**
	STDEV			
PFMPA	**Average**	**ND**	**ND**	**ND**
	STDEV			
PFBMA	**Average**	**ND**	**ND**	**ND**
	STDEV			
NFDHA	**Average**	**ND**	**ND**	**ND**
	STDEV			
PFEESA	**Average**	**ND**	**ND**	**ND**
	STDEV			

PF3ONS	**Average**	**ND**	**ND**	**ND**
	STDEV			
PF3OUdS	**Average**	**ND**	**ND**	**ND**
	STDEV			
**$\sum_{11}$PFCAs**		**31.7**	**71.2**	**89.8**
**$\sum_{8}$PFSAs**		**15.2**	**23.4**	**23.3**
**$\sum_{19}$PFAAs**		**46.9**	**94.7**	**113.1**
**$\sum_{7}$Short-chain PFAAs**		**28.5**	**69.8**	**93.0**
**$\sum_{12}$Long-chain PFAAs**		**18.5**	**24.8**	**20.1**
**$\sum_{21}$Precursors**		**66.4**	**3.4**	**3.1**
**$\sum_{40}$PFAS**		**113.3**	**98.1**	**116.2**

[a] Average value and standard deviation for the duplicate. [b]Limit of detection [c]Not detected

Table S7. PFAS concentrations (ng·L^{-1}) in wastewater samples in WWTP D (Sampling date: May 2022).

PFAS	ng/L	Primary INF	Activated Sludge EFF	Final EFF
PFBA	**Average**[a]	**3.8**	**9.2**	**8.8**
	STDEV[a]	0.7	1.2	1.9
PFPeA	**Average**	**3.3**	**6.5**	**6.2**
	STDEV	0.4	0.2	1.1
PFHxA	**Average**	**12.7**	**22.4**	**22.1**
	STDEV	1.2	1.0	2.3
PFHpA	**Average**	**15.3**	**12.9**	**10.5**
	STDEV	1.8	0.0	0.7
PFOA	**Average**	**8.1**	**11.2**	**8.9**
	STDEV	0.1	0.4	0.9
PFNA	**Average**	**2.0**	**0.7**	**0.7**
	STDEV	0.2	0.0	0.0
PFDA	**Average**	**4.8**	**0.8**	**0.7**
	STDEV	0.4	0.0	0.0
PFUdA	**Average**	**<LOD**[b]	**<LOD**	**<LOD**
	STDEV			
PFDoA	**Average**	**ND**[c]	**ND**	**ND**
	STDEV			
PFTrDA	**Average**	**ND**	**ND**	**ND**
	STDEV			
PFTeDA	**Average**	**ND**	**ND**	**ND**
	STDEV			
PFPrS	**Average**	**ND**	**ND**	**ND**
	STDEV			
PFBS	**Average**	**7.8**	**8.3**	**8.1**
	STDEV	0.1	0.0	0.3
PFPeS	**Average**	**<LOD**	**<LOD**	**<LOD**
	STDEV			
PFHxS	**Average**	**5.4**	**5.2**	**5.1**
	STDEV	0.1	0.3	0.4
PFHpS	**Average**	**0.4**	**0.3**	**0.3**
	STDEV	0.0	0.0	0.0
PFOS	**Average**	**9.8**	**10.2**	**11.1**
	STDEV	0.8	0.3	1.0
PFNS	**Average**	**<LOD**	**<LOD**	**<LOD**
	STDEV			

PFDS	**Average**	**<LOD**	**<LOD**	**<LOD**
	STDEV			
6:2 FTCA	**Average**	**0.7**	**<LOD**	**<LOD**
	STDEV	0.1		
6:2 FTUCA	**Average**	**<LOD**	**<LOD**	**<LOD**
	STDEV			
5:3 FTCA	**Average**	**8.5**	**<LOD**	**<LOD**
	STDEV	0.4		
4:2 FTS	**Average**	**<LOD**	**<LOD**	**<LOD**
	STDEV			
6:2 FTS	**Average**	**1.8**	**1.6**	**1.5**
	STDEV	0.1	0.1	0.1
8:2 FTS	**Average**	**<LOD**	**<LOD**	**<LOD**
	STDEV			
6:2 diPAP	**Average**	**21.7**	**7.2**	**10.8**
	STDEV	1.4	0.7	0.0
6:2/8:2 diPAP	**Average**	**<LOD**	**<LOD**	**<LOD**
	STDEV			
FOSA	**Average**	**<LOD**	**<LOD**	**<LOD**
	STDEV			
nMeFOSAA	**Average**	**<LOD**	**<LOD**	**<LOD**
	STDEV			
nEtFOSAA	**Average**	**<LOD**	**<LOD**	**<LOD**
	STDEV			
FBSA	**Average**	**ND**	**ND**	**ND**
	STDEV			
FHxSA	**Average**	**<LOD**	**<LOD**	**<LOD**
	STDEV			
HFPO-DA	**Average**	**34.9**	**43.3**	**55.2**
	STDEV	5.2	6.2	3.4
NaDONA	**Average**	**ND**	**ND**	**ND**
	STDEV			
PFMPA	**Average**	**ND**	**ND**	**ND**
	STDEV			
PFBMA	**Average**	**ND**	**ND**	**ND**
	STDEV			
NFDHA	**Average**	**ND**	**ND**	**ND**
	STDEV			
PFEESA	**Average**	**ND**	**ND**	**ND**
	STDEV			

PF3ONS	**Average**	**ND**	**ND**	**ND**
	STDEV			
PF3OUdS	**Average**	**ND**	**ND**	**ND**
	STDEV			
**$\sum_{11}$PFCAs**		**50.0**	**63.6**	**58.0**
**$\sum_{8}$PFSAs**		**23.5**	**24.0**	**24.6**
**$\sum_{19}$PFAAs**		**73.4**	**87.7**	**82.6**
**$\sum_{7}$Short-chain PFAAs**		**42.9**	**59.2**	**55.7**
**$\sum_{12}$Long-chain PFAAs**		**30.6**	**28.5**	**26.8**
**$\sum_{21}$Precursors**		**67.6**	**52.1**	**67.5**
**$\sum_{40}$PFAS**		**141.0**	**139.8**	**150.1**

[a] Average value and standard deviation for the duplicate. [b]Limit of detection [c]Not detected

Table S8. PFAS concentrations ($ng \cdot L^{-1}$) in wastewater samples in WWTP E (Sampling date: May 2022).

PFAS	ng/L	Primary INF	Activated Sludge EFF	Final EFF
PFBA	**Average**[a]	**1.6**	**4.7**	**12.2**
	STDEV[a]	0.1	0.0	0.7
PFPeA	**Average**	**13.2**	**15.1**	**15.3**
	STDEV	2.1	1.0	3.4
PFHxA	**Average**	**10.9**	**25.4**	**25.0**
	STDEV	0.2	0.4	0.7
PFHpA	**Average**	**5.7**	**9.7**	**4.9**
	STDEV	0.2	0.2	1.8
PFOA	**Average**	**17.8**	**24.8**	**18.7**
	STDEV	0.2	0.4	0.7
PFNA	**Average**	**2.1**	**1.6**	**1.5**
	STDEV	0.0	0.0	0.1
PFDA	**Average**	**4.5**	**1.8**	**2.3**
	STDEV	0.1	0.1	0.3
PFUdA	**Average**	**ND**[b]	**ND**	**ND**
	STDEV			
PFDoA	**Average**	**ND**	**ND**	**ND**
	STDEV			
PFTrDA	**Average**	**ND**	**ND**	**ND**
	STDEV			
PFTeDA	**Average**	**ND**	**ND**	**ND**
	STDEV			
PFPrS	**Average**	**ND**	**ND**	**ND**
	STDEV			
PFBS	**Average**	**5.0**	**6.4**	**5.4**
	STDEV	0.2	0.2	0.5
PFPeS	**Average**	**<LOD**[c]	**<LOD**	**<LOD**
	STDEV			
PFHxS	**Average**	**3.3**	**3.4**	**2.9**
	STDEV	0.2	0.1	0.1
PFHpS	**Average**	**0.4**	**0.3**	**0.2**
	STDEV	0.0	0.0	0.0
PFOS	**Average**	**26.1**	**24.7**	**15.8**
	STDEV	0.3	4.1	2.3
PFNS	**Average**	**<LOD**	**<LOD**	**<LOD**
	STDEV			

PFDS	**Average**	**<LOD**	**<LOD**	**<LOD**
	STDEV			
6:2 FTCA	**Average**	**0.8**	**0.1**	**<LOD**
	STDEV	0.1	0.0	
6:2 FTUCA	**Average**	**<LOD**	**<LOD**	**<LOD**
	STDEV			
5:3 FTCA	**Average**	**8.5**	**2.3**	**<LOD**
	STDEV	0.8	0.0	
4:2 FTS	**Average**	**<LOD**	**<LOD**	**<LOD**
	STDEV			
6:2 FTS	**Average**	**1.0**	**0.4**	**<LOD**
	STDEV	0.0	0.1	
8:2 FTS	**Average**	**<LOD**	**<LOD**	**<LOD**
	STDEV			
6:2 diPAP	**Average**	**15.1**	**11.6**	**7.1**
	STDEV	4.0	0.9	0.2
6:2/8:2 diPAP	**Average**	**<LOD**	**<LOD**	**<LOD**
	STDEV			
FOSA	**Average**	**<LOD**	**<LOD**	**<LOD**
	STDEV			
nMeFOSAA	**Average**	**<LOD**	**<LOD**	**<LOD**
	STDEV			
nEtFOSAA	**Average**	**<LOD**	**<LOD**	**<LOD**
	STDEV			
FBSA	**Average**	**<LOD**	**<LOD**	**<LOD**
	STDEV			
FHxSA	**Average**	**<LOD**	**<LOD**	**<LOD**
	STDEV			
HFPO-DA	**Average**	**35.3**	**35.7**	**32.2**
	STDEV	6.8	3.0	1.3
NaDONA	**Average**	**ND**	**ND**	**ND**
	STDEV			
PFMPA	**Average**	**ND**	**ND**	**ND**
	STDEV			
PFBMA	**Average**	**ND**	**ND**	**ND**
	STDEV			
NFDHA	**Average**	**ND**	**ND**	**ND**
	STDEV			
PFEESA	**Average**	**ND**	**ND**	**ND**
	STDEV			

PF3ONS	**Average**	**ND**	**ND**	**ND**
	STDEV			
PF3OUdS	**Average**	**ND**	**ND**	**ND**
	STDEV			
**$\sum_{11}$PFCAs**		**55.9**	**83.1**	**79.9**
**$\sum_{8}$PFSAs**		**34.8**	**34.8**	**24.3**
**$\sum_{19}$PFAAs**		**90.7**	**117.9**	**104.2**
**$\sum_{7}$Short-chain PFAAs**		**36.5**	**61.3**	**62.7**
**$\sum_{12}$Long-chain PFAAs**		**54.2**	**56.6**	**41.4**
**$\sum_{21}$Precursors**		**60.8**	**50.2**	**39.3**
**$\sum_{40}$PFAS**		**151.5**	**168.1**	**143.5**

[a] Average value and standard deviation for the duplicate. [b]Not detected [c]Limit of detection

Table S9. PFAS concentrations (ng·L^{-1}) in wastewater samples in WWTP F (Sampling date: April 2023).

PFAS	ng/L	Primary INF	Activated Sludge EFF	Final EFF
PFBA	**Average**[a]	**14.7**	**13.1**	**13.1**
	STDEV[a]	1.8	2.0	0.5
PFPeA	**Average**	**4.1**	**6.2**	**4.6**
	STDEV	0.5	0.6	0.2
PFHxA	**Average**	**3.1**	**5.8**	**7.5**
	STDEV	0.5	1.4	1.4
PFHpA	**Average**	**1.5**	**2.6**	**1.4**
	STDEV	0.4	0.2	0.4
PFOA	**Average**	**5.3**	**8.1**	**4.1**
	STDEV	0.1	0.3	0.4
PFNA	**Average**	**0.7**	**0.7**	**0.3**
	STDEV	0.1	0.1	0.0
PFDA	**Average**	**0.4**	**1.2**	**1.1**
	STDEV	0.2	0.4	0.6
PFUdA	**Average**	**2.0**	**3.1**	**<LOD**[b]
	STDEV	0.3	1.5	
PFDoA	**Average**	**ND**[c]	**ND**	**ND**
	STDEV			
PFTrDA	**Average**	**ND**	**ND**	**ND**
	STDEV			
PFTeDA	**Average**	**ND**	**ND**	**ND**
	STDEV			
PFPrS	**Average**	**1.4**	**1.5**	**0.5**
	STDEV	0.2	0.0	0.0
PFBS	**Average**	**3.7**	**3.7**	**3.8**
	STDEV	0.3	0.1	0.3
PFPeS	**Average**	**<LOD**	**<LOD**	**<LOD**
	STDEV			
PFHxS	**Average**	**0.7**	**1.4**	**0.9**
	STDEV	0.1	0.2	0.3
PFHpS	**Average**	**<LOD**	**<LOD**	**<LOD**
	STDEV			
PFOS	**Average**	**8.6**	**16.5**	**7.0**
	STDEV	0.1	2.3	1.7
PFNS	**Average**	**<LOD**	**<LOD**	**<LOD**
	STDEV			

PFDS	**Average**	**<LOD**	**<LOD**	**<LOD**
	STDEV			
6:2 FTCA	**Average**	**3.6**	**3.5**	**3.2**
	STDEV	0.2	0.4	0.4
6:2 FTUCA	**Average**	**25.9**	**7.6**	**7.1**
	STDEV	0.7	1.7	0.6
5:3 FTCA	**Average**	**4.2**	**4.9**	**1.3**
	STDEV	0.6	0.5	0.9
4:2 FTS	**Average**	**<LOD**	**<LOD**	**<LOD**
	STDEV			
6:2 FTS	**Average**	**19.5**	**12.6**	**16.4**
	STDEV	0.5	4.6	0.6
8:2 FTS	**Average**	**<LOD**	**<LOD**	**<LOD**
	STDEV			
6:2 diPAP	**Average**	**11.8**	**15.9**	**1.5**
	STDEV	2.2	1.8	0.0
6:2/8:2 diPAP	**Average**	**12.7**	**9.7**	**1.8**
	STDEV	3.3	2.2	0.1
FOSA	**Average**	**<LOD**	**<LOD**	**<LOD**
	STDEV			
nMeFOSAA	**Average**	**26.6**	**24.2**	**19.1**
	STDEV	3.1	2.1	0.4
nEtFOSAA	**Average**	**<LOD**	**<LOD**	**<LOD**
	STDEV			
FBSA	**Average**	**<LOD**	**<LOD**	**<LOD**
	STDEV			
FHxSA	**Average**	**<LOD**	**<LOD**	**<LOD**
	STDEV			
HFPO-DA	**Average**	**15.6**	**6.9**	**21.1**
	STDEV	0.1	0.2	1.5
NaDONA	**Average**	**9.1**	**15.8**	**12.0**
	STDEV	3.6	1.3	0.1
PFMPA	**Average**	**ND**	**ND**	**ND**
	STDEV			
PFBMA	**Average**	**ND**	**ND**	**ND**
	STDEV			
NFDHA	**Average**	**ND**	**ND**	**ND**
	STDEV			
PFEESA	**Average**	**ND**	**ND**	**ND**
	STDEV			

PF3ONS	**Average**	**ND**	**ND**	**ND**
	STDEV			
PF3OUdS	**Average**	**ND**	**ND**	**ND**
	STDEV			
**$\sum_{11}$PFCAs**		**31.8**	**40.9**	**32.1**
**$\sum_{8}$PFSAs**		**14.4**	**23.1**	**12.2**
**$\sum_{19}$PFAAs**		**46.2**	**64.0**	**44.2**
**$\sum_{7}$Short-chain PFAAs**		**28.6**	**32.9**	**30.8**
**$\sum_{12}$Long-chain PFAAs**		**17.9**	**31.1**	**13.4**
**$\sum_{21}$Precursors**		**128.2**	**101.0**	**83.7**
**$\sum_{40}$PFAS**		**174.4**	**165.0**	**127.9**

[a] Average value and standard deviation for the duplicate. [b]Limit of detection [c]Not detected

Table 10. PFAS concentrations (ng·L^{-1}) in wastewater samples in WWTP G (Sampling date: March 2023).

PFAS	ng/L	Primary INF	Activated Sludge EFF	Final EFF
PFBA	**Average**[a]	**0.6**	**1.7**	**1.5**
	STDEV[a]	0.0	0.4	0.0
PFPeA	**Average**	**29.8**	**37.7**	**20.0**
	STDEV	0.8	5.7	0.1
PFHxA	**Average**	**5.4**	**7.3**	**8.1**
	STDEV	0.1	1.3	1.5
PFHpA	**Average**	**1.0**	**5.0**	**1.8**
	STDEV	0.2	0.0	0.0
PFOA	**Average**	**9.8**	**16.2**	**10.8**
	STDEV	0.5	0.3	0.5
PFNA	**Average**	**0.3**	**1.5**	**0.4**
	STDEV	0.1	0.0	0.1
PFDA	**Average**	**4.4**	**11.0**	**4.0**
	STDEV	0.8	0.1	0.3
PFUdA	**Average**	**<LOD**[b]	**5.3**	**0.4**
	STDEV		0.4	0.1
PFDoA	**Average**	**ND**[c]	**ND**	**ND**
	STDEV			
PFTrDA	**Average**	**ND**	**ND**	**ND**
	STDEV			
PFTeDA	**Average**	**ND**	**ND**	**ND**
	STDEV			
PFPrS	**Average**	**<LOD**	**0.3**	**0.6**
	STDEV		0.5	0.1
PFBS	**Average**	**5.3**	**11.9**	**14.6**
	STDEV	0.4	0.2	0.2
PFPeS	**Average**	**<LOD**	**<LOD**	**<LOD**
	STDEV			
PFHxS	**Average**	**1.0**	**2.7**	**1.5**
	STDEV	0.3	0.4	0.1
PFHpS	**Average**	**<LOD**	**<LOD**	**<LOD**
	STDEV			
PFOS	**Average**	**6.9**	**10.9**	**6.7**
	STDEV	1.5	3.6	1.5
PFNS	**Average**	**<LOD**	**<LOD**	**<LOD**
	STDEV			

PFDS	**Average**	**<LOD**	**<LOD**	**<LOD**
	STDEV			
6:2 FTCA	**Average**	**<LOD**	**<LOD**	**<LOD**
	STDEV			
6:2 FTUCA	**Average**	**24.4**	**29.7**	**17.4**
	STDEV	9.0	11.2	9.4
5:3 FTCA	**Average**	**12.4**	**19.5**	**20.1**
	STDEV	6.7	0.8	4.1
4:2 FTS	**Average**	**<LOD**	**<LOD**	**<LOD**
	STDEV			
6:2 FTS	**Average**	**74.9**	**35.9**	**48.0**
	STDEV	63.8	11.9	0.2
8:2 FTS	**Average**	**<LOD**	**<LOD**	**<LOD**
	STDEV			
6:2 diPAP	**Average**	**<LOD**	**<LOD**	**<LOD**
	STDEV			
6:2/8:2 diPAP	**Average**	**<LOD**	**<LOD**	**<LOD**
	STDEV			
FOSA	**Average**	**<LOD**	**<LOD**	**1.6**
	STDEV			1.1
FBSA	**Average**	**<LOD**	**1.4**	**0.6**
	STDEV		1.4	0.0
FHxSA	**Average**	**<LOD**	**<LOD**	**<LOD**
	STDEV			
nMeFOSAA	**Average**	**<LOD**	**<LOD**	**<LOD**
	STDEV			
nEtFOSAA	**Average**	**<LOD**	**<LOD**	**<LOD**
	STDEV			
HFPO-DA	**Average**	**<LOD**	**<LOD**	**<LOD**
	STDEV			
NaDONA	**Average**	**5.5**	**19.3**	**5.3**
	STDEV	1.5	5.5	2.0
PFMPA	**Average**	**ND**	**ND**	**ND**
	STDEV			
PFBMA	**Average**	**ND**	**ND**	**ND**
	STDEV			
NFDHA	**Average**	**ND**	**ND**	**ND**
	STDEV			
PFEESA	**Average**	**ND**	**ND**	**ND**
	STDEV			

PF3ONS	**Average**<br>STDEV	**ND**	**ND**	**ND**
PF3OUdS	**Average**<br>STDEV	**ND**	**ND**	**ND**
**∑$_{11}$PFCAs**		**51.3**	**85.8**	**47.0**
**∑$_{8}$PFSAs**		**13.1**	**45.9**	**23.4**
**∑$_{19}$PFAAs**		**64.4**	**131.6**	**70.4**
**∑$_{7}$Short-chain PFAAs**		**42.1**	**64.0**	**46.6**
**∑$_{12}$Long-chain PFAAs**		**22.3**	**67.6**	**23.8**
**∑$_{21}$Precursors**		**117.2**	**105.9**	**93.0**
**∑$_{40}$PFAS**		**181.6**	**237.5**	**163.4**

[a]Average value and standard deviation for the duplicate. [b]Limit of detection limit [c]Not detected

Table S11. PFAS concentrations (ng·L^{-1}) in wastewater samples in WWTP H (Sampling date: November 2022).

PFAS	ng/L	Primary INF	Activated Sludge EFF	Final EFF
PFBA	**Average**	**<MDL[b]**	**<MDL**	**<MDL**
	STDEV			
PFPeA	**Average**	**7.6**	**15.1**	**9.5**
	STDEV	0.9	1.5	0.8
PFHxA	**Average**	**6.0**	**12.7**	**11.6**
	STDEV	0.8	0.8	0.7
PFHpA	**Average**	**3.1**	**1.4**	**1.1**
	STDEV	0.4	0.0	0.1
PFOA	**Average**	**9.6**	**9.7**	**8.6**
	STDEV	0.7	0.5	0.4
PFNA	**Average**	**0.9**	**0.3**	**0.3**
	STDEV	0.1	0.1	0.0
PFDA	**Average**	**6.7**	**7.4**	**5.5**
	STDEV	0.0	0.1	0.1
PFUdA	**Average**	**ND[c]**	**ND**	**ND**
	STDEV			
PFDoA	**Average**	**ND**	**ND**	**ND**
	STDEV			
PFTrDA	**Average**	**ND**	**ND**	**ND**
	STDEV			
PFTeDA	**Average**	**ND**	**ND**	**ND**
	STDEV			
PFPrS	**Average**	**2.0**	**<LOD**	**<LOD**
	STDEV	0.4		
PFBS	**Average**	**4.2**	**4.8**	**4.0**
	STDEV	0.2	0.0	0.1
PFPeS	**Average**	**<LOD[d]**	**<LOD**	**<LOD**
	STDEV			
PFHxS	**Average**	**1.8**	**1.3**	**1.3**
	STDEV	0.1	0.2	0.2
PFHpS	**Average**	**<LOD**	**<LOD**	**<LOD**
	STDEV			
PFOS	**Average**	**12.0**	**12.0**	**7.6**
	STDEV	2.7	0.0	1.0
PFNS	**Average**	**<LOD**	**<LOD**	**<LOD**
	STDEV			

PFDS	**Average**	**<LOD**	**<LOD**	**<LOD**
	STDEV			
6:2 FTCA	**Average**	**1.9**	**0.4**	**0.8**
	STDEV	0.7	0.0	0.0
6:2 FTUCA	**Average**	**2.3**	**1.7**	**1.4**
	STDEV	0.8	0.2	0.0
5:3 FTCA	**Average**	**70.1**	**22.9**	**17.3**
	STDEV	1.9	3.2	0.3
4:2 FTS	**Average**	**<LOD**	**<LOD**	**<LOD**
	STDEV			
6:2 FTS	**Average**	**41.5**	**35.5**	**27.4**
	STDEV	8.7	3.9	0.5
8:2 FTS	**Average**	**<LOD**	**<LOD**	**<LOD**
	STDEV			
6:2 diPAP	**Average**	**11.8**	**0.7**	**1.0**
	STDEV	2.1	0.0	0.2
6:2/8:2 diPAP	**Average**	**11.1**	**<LOD**	**<LOD**
	STDEV	0.4		
FOSA	**Average**	**9.4**	**0.2**	**0.5**
	STDEV	0.2	0.0	0.1
nMeFOSAA	**Average**	**47.4**	**30.4**	**30.0**
	STDEV	8.2	2.9	0.2
nEtFOSAA	**Average**	**5.3**	**<LOD**	**<LOD**
	STDEV	1.1		
FBSA	**Average**	**20.4**	**<LOD**	**<LOD**
	STDEV	1.3		
FHxSA	**Average**	**<LOD**	**<LOD**	**<LOD**
	STDEV			
HFPO-DA	**Average**	**5.4**	**8.8**	**4.7**
	STDEV	0.1	1.1	0.2
NaDONA	**Average**	**ND**	**ND**	**ND**
	STDEV			
PFMPA	**Average**	**ND**	**ND**	**ND**
	STDEV			
PFBMA	**Average**	**ND**	**ND**	**ND**
	STDEV			
NFDHA	**Average**	**ND**	**ND**	**ND**
	STDEV			
PFEESA	**Average**	**ND**	**ND**	**ND**
	STDEV			

PF3ONS	**Average**	**ND**	**ND**	**ND**
	STDEV			
PF3OUdS	**Average**	**ND**	**ND**	**ND**
	STDEV			
**$\sum_{11}$PFCAs**		**33.9**	**46.6**	**36.7**
**$\sum_{8}$PFSAs**		**20.1**	**18.1**	**12.9**
**$\sum_{19}$PFAAs**		**53.9**	**64.7**	**49.7**
**$\sum_{7}$Short-chain PFAAs**		**23.0**	**34.0**	**26.3**
**$\sum_{12}$Long-chain PFAAs**		**30.9**	**30.8**	**23.4**
**$\sum_{21}$Precursors**		**226.6**	**100.6**	**83.3**
**$\sum_{40}$PFAS**		**280.5**	**165.3**	**133.0**

[a] Average value and standard deviation for the duplicate. [b]Method detection limit [c]Not detected [d]Limit of detection

Table S12. PFAS concentrations ($ng \cdot L^{-1}$) in wastewater samples in WWTP I (Sampling date: February 2023).

PFAS	ng/L	Primary INF	Activated Sludge EFF	Final EFF
PFBA	**Average**[a]	**13.4**	**13.8**	**13.2**
	STDEV[a]	3.6	0.2	0.8
PFPeA	**Average**	**5.2**	**12.4**	**16.5**
	STDEV	1.0	1.1	2.9
PFHxA	**Average**	**5.4**	**14.1**	**16.0**
	STDEV	0.2	2.4	1.5
PFHpA	**Average**	**4.6**	**2.6**	**2.4**
	STDEV	0.5	1.0	0.4
PFOA	**Average**	**18.9**	**27.4**	**23.6**
	STDEV	1.0	1.2	6.4
PFNA	**Average**	**<LOD**[b]	**0.6**	**0.4**
	STDEV		0.1	0.2
PFDA	**Average**	**<LOD**	**3.3**	**2.9**
	STDEV		0.4	0.3
PFUdA	**Average**	**<LOD**	**0.7**	**0.5**
	STDEV		0.2	0.0
PFDoA	**Average**	**ND**[c]	**ND**	**ND**
	STDEV			
PFTrDA	**Average**	**ND**	**ND**	**ND**
	STDEV			
PFTeDA	**Average**	**ND**	**ND**	**ND**
	STDEV			
PFPrS	**Average**	**ND**	**ND**	**ND**
	STDEV			
PFBS	**Average**	**7.9**	**7.3**	**2.7**
	STDEV	0.3	0.2	0.4
PFPeS	**Average**	**<LOD**	**<LOD**	**<LOD**
	STDEV			
PFHxS	**Average**	**4.5**	**4.8**	**1.2**
	STDEV	0.5	0.5	0.1
PFHpS	**Average**	**0.4**	**0.3**	**<LOD**
	STDEV	0.1	0.1	
PFOS	**Average**	**19.4**	**19.9**	**13.3**
	STDEV	3.5	0.5	0.1
PFNS	**Average**	**<LOD**	**<LOD**	**<LOD**
	STDEV			

PFDS	**Average**	**<LOD**	**<LOD**	**<LOD**
	STDEV			
6:2 FTCA	**Average**	**<LOD**	**<LOD**	**<LOD**
	STDEV			
6:2 FTUCA	**Average**	**21.4**	**8.5**	**4.4**
	STDEV	11.7	4.8	3.9
5:3 FTCA	**Average**	**42.4**	**<LOD**	**<LOD**
	STDEV	6.4	**<LOD**	**<LOD**
4:2 FTS	**Average**	**22.8**	**12.0**	**14.8**
	STDEV	8.4	2.1	5.5
6:2 FTS	**Average**	**70.2**	**63.2**	**46.6**
	STDEV	6.5	5.7	8.1
8:2 FTS	**Average**	**<LOD**	**<LOD**	**<LOD**
	STDEV			
6:2 diPAP	**Average**	**1.8**	**0.4**	**0.8**
	STDEV	0.1	0.6	0.3
6:2/8:2 diPAP	**Average**	**1.2**	**<LOD**	**<LOD**
	STDEV	0.2		
FOSA	**Average**	**<LOD**	**<LOD**	**1.6**
	STDEV			1.1
FBSA	**Average**	**1.4**	**0.5**	**0.3**
	STDEV	0.4	0.1	0.3
FHxSA	**Average**	**<LOD**	**<LOD**	**<LOD**
	STDEV			
nMeFOSAA	**Average**	**84.9**	**35.2**	**29.3**
	STDEV	12.1	2.0	3.8
nEtFOSAA	**Average**	**<LOD**	**<LOD**	**<LOD**
	STDEV			
HFPO-DA	**Average**	**14.8**	**<LOD**	**1.5**
	STDEV	3.9		0.2
NaDONA	**Average**	**5.5**	**7.8**	**5.3**
	STDEV	1.5	0.3	2.0
PFMPA	**Average**	**ND**	**ND**	**ND**
	STDEV			
PFBMA	**Average**	**ND**	**ND**	**ND**
	STDEV			
NFDHA	**Average**	**ND**	**ND**	**ND**
	STDEV			
PFEESA	**Average**	**ND**	**ND**	**ND**
	STDEV			

PF3ONS	**Average**	**ND**	**ND**	**ND**
	STDEV			
PF3OUdS	**Average**	**ND**	**ND**	**ND**
	STDEV			
**$\sum_{11}$PFCAs**		**47.5**	**74.9**	**75.4**
**$\sum_{8}$PFSAs**		**32.2**	**32.3**	**17.3**
**$\sum_{19}$PFAAs**		**79.7**	**107.2**	**92.7**
**$\sum_{7}$Short-chain PFAAs**		**36.5**	**50.2**	**40.9**
**$\sum_{12}$Long-chain PFAAs**		**43.2**	**57.0**	**41.9**
**$\sum_{21}$Precursors**		**266.5**	**128.3**	**104.0**
**$\sum_{40}$PFAS**		**346.2**	**235.5**	**196.7**

[a]Average value and standard deviation for the duplicate. [b] Limit of detection [c]Not detected

Table S13. PFCA concentrations ($ng \cdot L^{-1}$) in wastewater samples in WWTP B after TOP oxidation.

PFAS	ng/L	INF	AS EFF	EFF
PFBA	**Average** [a]	**32.2**	**17.7**	**18.3**
	STDEV [a]	0.1	0.3	1.1
PFPeA	**Average**	**45.4**	**29.9**	**27.5**
	STDEV	1.0	0.4	0.7
PFHxA	**Average**	**24.9**	**18.2**	**17.1**
	STDEV	1.8	1.0	1.2
PFHpA	**Average**	**13.6**	**2.9**	**2.4**
	STDEV	0.3	0.1	0.4
PFOA	**Average**	**38.2**	**22.5**	**18.7**
	STDEV	4.1	6.6	0.1
PFNA	**Average**	**5.2**	**1.4**	**1.1**
	STDEV	0.0	0.1	0.1
PFDA	**Average**	**4.9**	**3.7**	**1.9**
	STDEV	0.9	0.1	0.2
PFUdA	**Average**	**ND** [b]	**ND**	**ND**
	STDEV			
PFDoA	**Average**	**ND**	**ND**	**ND**
	STDEV			
**∑Short-chain PFCAs**		**116.1**	**68.8**	**65.3**
**∑Long-chain PFCAs**		**48.4**	**27.6**	**21.6**
**∑PFCAs**		**164.5**	**96.3**	**86.9**
**ΔTOP[c], Short-chain PFCAs**		**100.3**	**20.0**	**25.7**
**ΔTOP, Long-chain PFCAs**		**40.7**	**15.7**	**13.5**
**ΔTOP, PFCAs**		**141.0**	**35.7**	**39.2**

[a] Average value and standard deviation for the duplicate.

[b] Not detected

[c] The increase of ΣPFCAs after TOP oxidation.

Table S14. PFCA concentrations (ng·L^{-1}) in wastewater samples in WWTP C after TOP oxidation.

PFAS	ng/L	INF	AS EFF	EFF
PFBA	**Average** [a]	**47.6**	**18.0**	**15.7**
	STDEV [a]	0.32	0.1	0.0
PFPeA	**Average**	**47.6**	**53.0**	**35.4**
	STDEV	3.1	1.6	0.3
PFHxA	**Average**	**33.6**	**26.9**	**32.3**
	STDEV	1.5	1.2	0.7
PFHpA	**Average**	**13.4**	**3.4**	**3.4**
	STDEV	1.0	0.1	0.5
PFOA	**Average**	**29.8**	**13.4**	**13.0**
	STDEV	0.4	0.2	0.2
PFNA	**Average**	**4.7**	**1.3**	**0.9**
	STDEV	0.0	0.0	0.1
PFDA	**Average**	**5.0**	**2.7**	**1.1**
	STDEV	0.8	0.6	0.1
PFUdA	**Average**	**ND**[b]	**ND**	**ND**
	STDEV			
PFDoA	**Average**	**ND**	**ND**	**ND**
	STDEV			
**∑Short-chain PFCAs**		**142.2**	**101.3**	**86.9**
**∑Long-chain PFCAs**		**39.4**	**17.4**	**15.0**
**∑PFCAs**		**181.6**	**118.7**	**101.9**
**ΔTOP[c], $_{\text{Short-chain PFCAs}}$**		**118.7**	**43.8**	**9.9**
**ΔTOP, $_{\text{Long-chain PFCAs}}$**		**31.2**	**3.7**	**2.1**
**ΔTOP, $_{\text{PFCAs}}$**		**149.9**	**47.5**	**12.1**

[a] Average value and standard deviation for the duplicate.

[b] Not detected

[c] The increase of ΣPFCAs after TOP oxidation.

Table S15. PFCA concentrations (ng·L^{-1}) in wastewater samples in WWTP D after TOP oxidation.

PFAS	ng/L	INF	AS EFF	EFF
PFBA	**Average** [a]	**26.0**	**17.6**	**11.1**
	STDEV [a]	1.5	0.0	0.8
PFPeA	**Average**	**46.8**	**25.0**	**18.8**
	STDEV	1.8	0.0	0.0
PFHxA	**Average**	**37.5**	**26.3**	**25.4**
	STDEV	4.0	1.3	1.5
PFHpA	**Average**	**29.8**	**14.5**	**15.5**
	STDEV	4.5	1.1	4.3
PFOA	**Average**	**18.2**	**15.1**	**16.2**
	STDEV	2.1	4.6	4.4
PFNA	**Average**	**5.0**	**2.6**	**2.6**
	STDEV	0.3	0.2	0.2
PFDA	**Average**	**5.4**	**1.3**	**1.0**
	STDEV	0.8	0.1	0.0
PFUdA	**Average**	**1.0**	**0.4**	**0.3**
	STDEV	0.0	0.1	0.0
PFDoA	**Average**	**1.4**	**ND** [b]	**ND**
	STDEV	0.1		
**∑Short-chain PFCAs**		**140.1**	**83.4**	**70.7**
**∑Long-chain PFCAs**		**31.0**	**19.4**	**20.1**
**∑PFCAs**		**171.1**	**102.8**	**90.8**
**ΔTOP[c], Short-chain PFCAs**		**105.0**	**32.5**	**23.1**
**ΔTOP, Long-chain PFCAs**		**16.1**	**6.6**	**9.7**
**ΔTOP, PFCAs**		**121.1**	**39.2**	**32.8**

[a] Average value and standard deviation for the duplicate.

[b] Not detected

[c] The increase of ΣPFCAs after TOP oxidation.

Table S16. PFCA concentrations ($ng \cdot L^{-1}$) in wastewater samples in WWTP E after TOP oxidation.

PFAS	ng/L	INF	AS EFF	EFF
PFBA	**Average** [a]	**18.8**	**12.2**	**11.3**
	STDEV [a]	1.9	0.4	1.8
PFPeA	**Average**	**29.9**	**15.6**	**15.9**
	STDEV	0.9	0.6	0.0
PFHxA	**Average**	**39.4**	**24.7**	**26.5**
	STDEV	6.2	3.2	1.6
PFHpA	**Average**	**10.4**	**10.1**	**9.3**
	STDEV	0.5	1.5	1.1
PFOA	**Average**	**37.5**	**30.5**	**25.1**
	STDEV	3.2	1.1	0.0
PFNA	**Average**	**3.7**	**1.7**	**1.7**
	STDEV	1.4	0.0	0.1
PFDA	**Average**	**7.2**	**2.0**	**3.7**
	STDEV	0.4	0.0	0.8
PFUdA	**Average**	**0.6**	**0.2**	**0.2**
	STDEV	0.3	0.0	0.0
PFDoA	**Average**	**0.5**	**ND** [b]	**ND**
	STDEV	0.3		
**∑Short-chain PFCAs**		**98.5**	**62.7**	**63.0**
**∑Long-chain PFCAs**		**49.5**	**34.5**	**30.7**
**∑PFCAs**		**148.1**	**97.2**	**93.7**
**ΔTOP[c], $_{\text{Short-chain PFCAs}}$**		**67.1**	**7.8**	**5.7**
**ΔTOP, $_{\text{Long-chain PFCAs}}$**		**25.1**	**6.3**	**8.2**
**ΔTOP, $_{\text{PFCAs}}$**		**92.2**	**14.1**	**13.9**

[a] Average value and standard deviation for the duplicate.

[b] Not detected

[c] The increase of ΣPFCAs after TOP oxidation.

Table S17. PFCA concentrations (ng·L^{-1}) in wastewater samples in WWTP F after TOP oxidation.

PFAS	ng/L	INF	AS EFF	EFF
PFBA	**Average**[a]	**20.2**	**15.4**	**14.6**
	STDEV[a]	1.5	2.4	4.1
PFPeA	**Average**	**24.3**	**14.8**	**12.5**
	STDEV	0.8	0.1	2.7
PFHxA	**Average**	**18.1**	**12.3**	**11.7**
	STDEV	0.1	0.0	0.4
PFHpA	**Average**	**6.4**	**1.8**	**1.6**
	STDEV	0.0	0.0	0.3
PFOA	**Average**	**14.1**	**11.7**	**11.0**
	STDEV	0.5	0.2	0.9
PFNA	**Average**	**2.1**	**0.8**	**0.3**
	STDEV	0.3	0.2	0.0
PFDA	**Average**	**10.3**	**11.1**	**10.7**
	STDEV	0.7	1.5	0.5
PFUdA	**Average**	**2.7**	**3.8**	**<LOD**[a]
	STDEV	0.0	0.4	
PFDoA	**Average**	**0.3**	**ND**[b]	**ND**
	STDEV	0.0		
**∑Short-chain PFCAs**		**71.4**	**46.2**	**42.6**
**∑Long-chain PFCAs**		**29.6**	**27.5**	**22.1**
**∑PFCAs**		**98.6**	**71.7**	**62.6**
**ΔTOP[d], Short-chain PFCAs**		**46.6**	**16.5**	**14.0**
**ΔTOP, Long-chain PFCAs**		**21.2**	**14.3**	**16.6**
**ΔTOP, PFCAs**		**66.8**	**30.8**	**30.6**

[a] Average value and standard deviation for the duplicate.

[b] Limit of detection

[c] Not detected

[d] The increase of ΣPFCAs after TOP oxidation.

Table S18. PFCA concentrations (ng·L^{-1}) in wastewater samples in WWTP G after TOP oxidation.

PFAS	ng/L	INF	AS EFF	EFF
PFBA	**Average[a]**	**28.3**	**14.0**	**12.1**
	STDEV[a]	1.6	1.7	0.8
PFPeA	**Average**	**43.1**	**39.8**	**22.3**
	STDEV	1.4	0.2	4.8
PFHxA	**Average**	**33.4**	**22.6**	**21.7**
	STDEV	0.2	0.0	0.8
PFHpA	**Average**	**12.3**	**6.2**	**3.0**
	STDEV	0.1	0.1	0.6
PFOA	**Average**	**27.0**	**22.3**	**19.7**
	STDEV	1.0	0.5	0.1
PFNA	**Average**	**3.7**	**1.5**	**0.6**
	STDEV	0.6	0.3	0.0
PFDA	**Average**	**18.6**	**20.0**	**12.2**
	STDEV	1.2	2.7	1.1
PFUdA	**Average**	**0.7**	**5.5**	**0.6**
	STDEV	0.1	0.5	0.0
PFDoA	**Average**	**ND[b]**	**ND**	**ND**
	STDEV			
**∑Short-chain PFCAs**		**117.1**	**82.6**	**59.1**
**∑Long-chain PFCAs**		**50.1**	**49.3**	**33.1**
**∑PFCAs**		**167.2**	**131.9**	**92.2**
**ΔTOP[d], $_{\text{Short-chain PFCAs}}$**		**81.4**	**30.9**	**27.6**
**ΔTOP, $_{\text{Long-chain PFCAs}}$**		**50.8**	**7.6**	**35.1**
**ΔTOP, $_{\text{PFCAs}}$**		**132.2**	**38.5**	**62.7**

[a] Average value and standard deviation for the duplicate.

[b] Limit of detection

[c] Not detected

[d] The increase of ΣPFCAs after TOP oxidation.

Table S19. PFCA concentrations (ng·L^{-1}) in wastewater samples in WWTP H after TOP oxidation.

PFAS	ng/L	INF	AS EFF	EFF
PFBA	**Average**[a]	**14.8**	**7.3**	**6.3**
	STDEV[a]	0.8	0.9	0.4
PFPeA	**Average**	**25.2**	**15.3**	**13.0**
	STDEV	0.8	0.1	2.8
PFHxA	**Average**	**18.6**	**12.6**	**12.1**
	STDEV	0.1	0.0	0.4
PFHpA	**Average**	**6.5**	**1.8**	**1.6**
	STDEV	0.0	0.0	0.3
PFOA	**Average**	**15.7**	**13.0**	**12.2**
	STDEV	0.6	0.3	1.0
PFNA	**Average**	**2.2**	**0.4**	**0.3**
	STDEV	0.3	0.0	0.0
PFDA	**Average**	**11.0**	**11.8**	**11.4**
	STDEV	0.7	1.6	0.5
PFUdA	**Average**	**0.4**	**0.1**	**0.1**
	STDEV	0.0	0.0	0.0
PFDoA	**Average**	**0.3**	**ND**[b]	**ND**
	STDEV	0.0		
**∑Short-chain PFCAs**		**65.1**	**37.1**	**33.0**
**∑Long-chain PFCAs**		**29.7**	**25.3**	**24.1**
**∑PFCAs**		**94.8**	**62.4**	**57.1**
**ΔTOP[d], $_{\text{Short-chain PFCAs}}$**		**48.3**	**8.2**	**11.3**
**ΔTOP, $_{\text{Long-chain PFCAs}}$**		**11.9**	**12.9**	**14.2**
**ΔTOP, $_{\text{PFCAs}}$**		**60.2**	**21.1**	**25.5**

[a] Average value and standard deviation for the duplicate.

[b] Limit of detection

[c] Not detected

[d] The increase of ΣPFCAs after TOP oxidation.

Table S20. PFCA concentrations ($ng \cdot L^{-1}$) in wastewater samples in WWTP I after TOP oxidation.

PFAS	ng/L	INF	AS EFF	EFF
PFBA	**Average** [a]	**28.3**	**14.0**	**13.3**
	STDEV [a]	1.6	1.7	1.0
PFPeA	**Average**	**43.1**	**26.2**	**22.3**
	STDEV	1.4	0.2	4.8
PFHxA	**Average**	**33.4**	**22.6**	**21.7**
	STDEV	0.2	0.0	0.8
PFHpA	**Average**	**12.3**	**3.5**	**3.0**
	STDEV	0.1	0.1	0.6
PFOA	**Average**	**27.0**	**22.3**	**21.0**
	STDEV	1.0	0.5	1.7
PFNA	**Average**	**3.7**	**0.6**	**0.6**
	STDEV	0.6	0.0	0.0
PFDA	**Average**	**18.6**	**20.0**	**19.3**
	STDEV	1.2	2.7	0.8
PFUdA	**Average**	**0.7**	**0.1**	**0.1**
	STDEV	0.1	0.0	0.0
PFDoA	**Average**	**ND** [b]	**ND**	**ND**
	STDEV			
**∑Short-chain PFCAs**		**117.1**	**66.3**	**60.3**
**∑Long-chain PFCAs**		**50.1**	**43.1**	**41.1**
**∑PFCAs**		**167.2**	**109.4**	**101.4**
**ΔTOP** [c]**, Short-chain PFCAs**		**89.4**	**24.2**	**13.8**
**ΔTOP, Long-chain PFCAs**		**32.9**	**19.5**	**25.5**
**ΔTOP, PFCAs**		**122.4**	**43.7**	**39.3**

[a] Average value and standard deviation for the duplicate.

[b] Not detected

[c] The increase of ΣPFCAs after TOP oxidation.

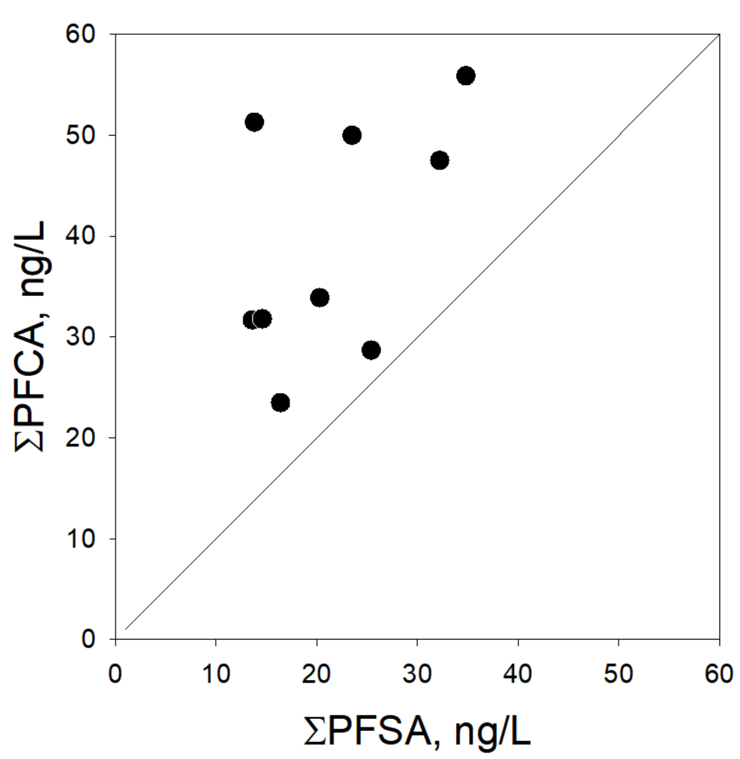

Figure S1. Relationship between summed PFSAs and PFCAs in municipal wastewater entering into nine WWTPs. Solid line is 1:1 ratio.

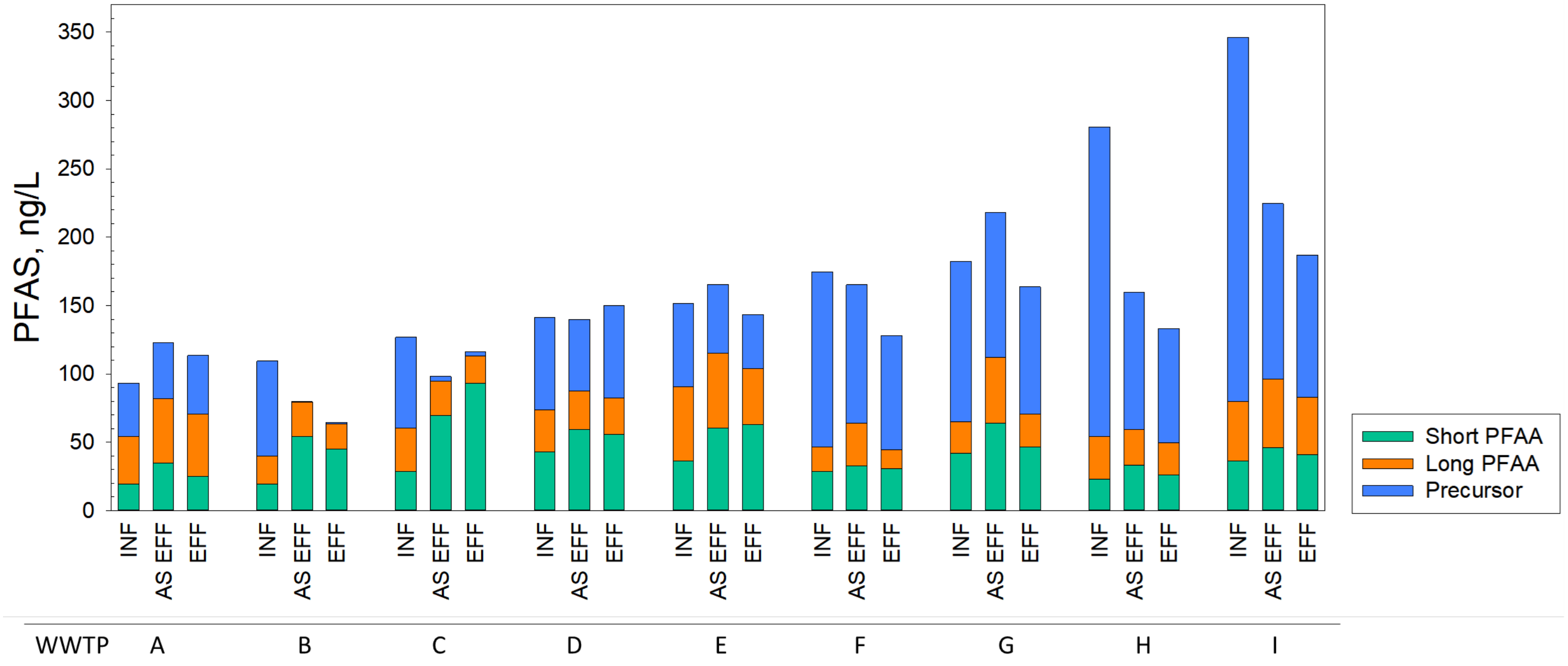

Figure S2. Concentrations of short-chain PFAAs (PFCAs < C8, PFSAs < C6), long-chain PFAAs and precursors in influents (INF), activated sludge effluent (AS EFF), and final effluent (EFF) in nine WWTPs.

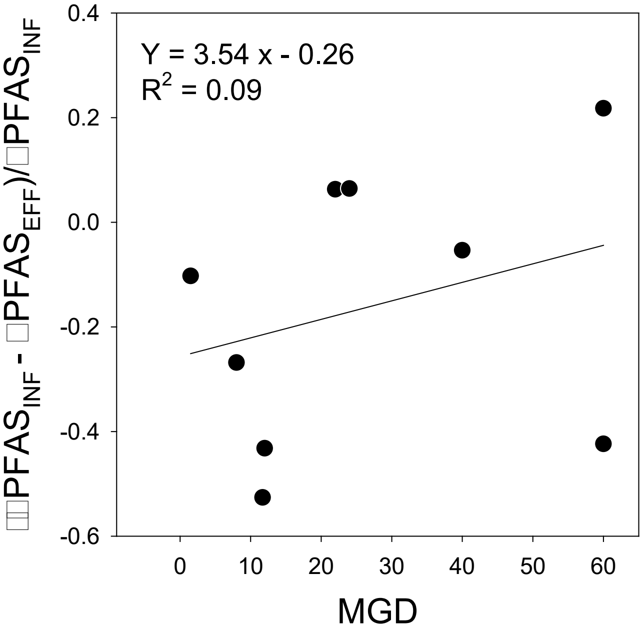

Figure S3. Relationship between MGD of WWTPs and PFAS removal efficiency ($\sum PFAS_{INF}$-$\sum PFAS_{EFF}$)/($\sum PFAS_{INF}$). Solid line is a linear regression line.

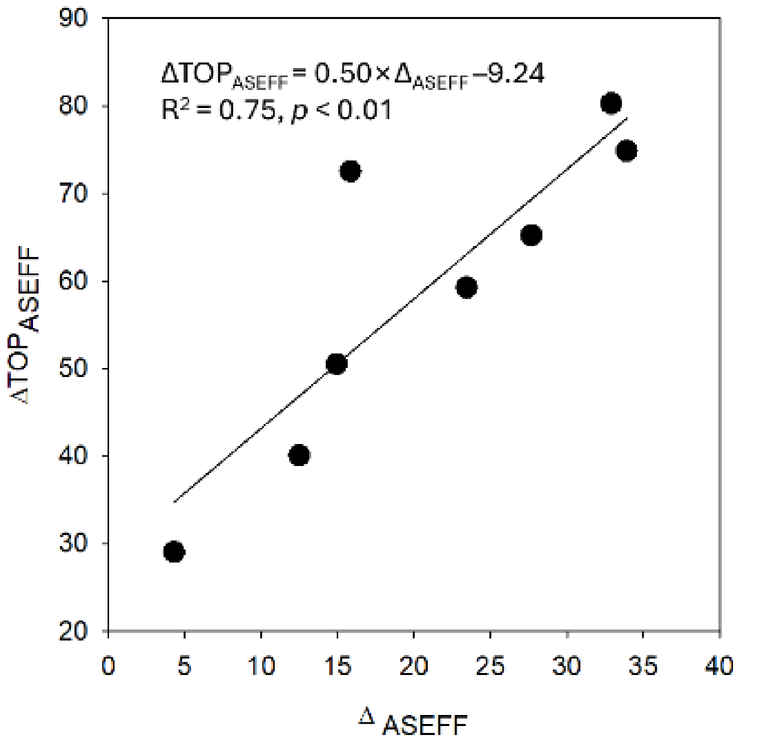

Figure S4. Relationship between the increase of ∑short-chain PFCAs after activated sludge treatment (expressed as Δ_{ASEFF}) and the decrease of ΔTOP of short-chain PFCAs after activated sludge treatment (expressed as ΔTOP_{ASEFF}).

References:

(1) Kim, J.; Xin, X.; Mamo, B. T.; Hawkins, G. L.; Li, K.; Chen, Y.; Huang, Q.; Huang, C.-H., Occurrence and fate of ultrashort-chain and other per-and polyfluoroalkyl substances (PFAS) in wastewater treatment plants. *ACS ES&T Water* **2022,** *2* (8), 1380-1390.
